# Mathematical Modeling of the Transmission Dynamics of *Clostridium difficile* Infection and Colonization in Healthcare Settings: A Systematic Review

**DOI:** 10.1371/journal.pone.0163880

**Published:** 2016-09-30

**Authors:** Guillaume Gingras, Marie-Hélène Guertin, Jean-François Laprise, Mélanie Drolet, Marc Brisson

**Affiliations:** 1 SP-POS, Centre de recherche du CHU de Québec-Université Laval, 1050 Chemin Sainte-Foy, Québec, Qc, Canada; 2 Départment de Médecine Sociale et Préventive, Université Laval, Québec, Qc, Canada; 3 Department of Infectious Disease Epidemiology, Imperial College, London, United Kingdom; University of Arizona, UNITED STATES

## Abstract

**Background:**

We conducted a systematic review of mathematical models of transmission dynamic of *Clostridium difficile* infection (CDI) in healthcare settings, to provide an overview of existing models and their assessment of different CDI control strategies.

**Methods:**

We searched MEDLINE, EMBASE and Web of Science up to February 3, 2016 for transmission-dynamic models of *Clostridium difficile* in healthcare settings. The models were compared based on their natural history representation of *Clostridium difficile*, which could include health states (S-E-A-I-R-D: Susceptible-Exposed-Asymptomatic-Infectious-Resistant-Deceased) and the possibility to include healthcare workers and visitors (vectors of transmission). Effectiveness of interventions was compared using the relative reduction (compared to no intervention or current practice) in outcomes such as incidence of colonization, CDI, CDI recurrence, CDI mortality, and length of stay.

**Results:**

Nine studies describing six different models met the inclusion criteria. Over time, the models have generally increased in complexity in terms of natural history and transmission dynamics and number/complexity of interventions/bundles of interventions examined. The models were categorized into four groups with respect to their natural history representation: S-A-I-R, S-E-A-I, S-A-I, and S-E-A-I-R-D. Seven studies examined the impact of CDI control strategies. Interventions aimed at controlling the transmission, lowering CDI vulnerability and reducing the risk of recurrence/mortality were predicted to reduce CDI incidence by 3–49%, 5–43% and 5–29%, respectively. Bundles of interventions were predicted to reduce CDI incidence by 14–84%.

**Conclusions:**

Although CDI is a major public health problem, there are very few published transmission-dynamic models of *Clostridium difficile*. Published models vary substantially in the interventions examined, the outcome measures used and the representation of the natural history of *Clostridium difficile*, which make it difficult to synthesize results and provide a clear picture of optimal intervention strategies. Future modeling efforts should pay specific attention to calibration, structural uncertainties, and transparent reporting practices.

## Introduction

*Clostridium difficile* (*C. difficile*) is a major public health problem that directly affects patient safety and disrupts hospital operations, causing significant health and economic consequences to the healthcare system [1–3]. *C. difficile* is an endospore-forming bacterium, which is spread mainly through the fecal-oral route. *C. difficile* can colonize the small intestine and the colon following the disturbance of the gut flora, typically caused by the exposure to antimicrobials. Proliferation of *C. difficile* can cause a broad range of clinical manifestations, varying from asymptomatic carriage, to diarrhea (*C. difficile* infection (CDI)), to pseudomembranous colitis and, in some cases, death. The greatest incidence of CDI is among individuals exposed to antimicrobial therapy, proton-pumps inhibitors and the elderly with a past medical history of hospitalization [4]. In hospital settings, three main pathways of *C. difficile* transmission have been documented: 1) contacts with infectious patients (asymptomatically colonized and symptomatic) [5, 6]; 2) exposure following contacts with healthcare workers (HCW) [7]; and 3) exposure caused by the environmental contamination [8, 9].

Over the past two decades, the incidence and severity of CDI and its related health care costs have increased dramatically in high income countries, mainly because of the emergence of a more virulent strain (BI/NAP1/027) [10]. Fortunately, new technologies and control strategies (e.g., rapid diagnostic tests [11], vaccines [12], introduction of antimicrobial stewardship programs [13–15] and enhanced infection control practices [16]) offer tremendous promise for the reduction of hospital-associated CDI. However, randomized clinical trials examining the optimal use and the combinations of these strategies are lacking because of: 1) their prohibitive costs; 2) challenges in comparing and isolating the benefit of multiple interventions within one study; 3) ethical issues (e.g., randomizing patients into experimental groups during outbreaks); and 4) limited generalizability because of differences in patient populations, hospital characteristics, and antimicrobial use can have an important impact on study findings. Hence, evidence on the efficacy of individual interventions or groups of interventions (bundles) are based mostly on observational studies [17] and remain very limited.

Mathematical models of infectious disease transmission dynamics have proven extremely valuable to address questions that are unfeasible or unethical in a clinical trial setting [18–20]. For healthcare-associated CDI, modeling can provide a formal framework to test and compare the effectiveness and cost-effectiveness of a large number of prevention and control strategies considering different hospital/patient characteristics and to address many possible assumptions about the natural history of *C. difficile*. However, predicting the impact of interventions against communicable diseases is particularly challenging since prevention, treatment or contact precautions in an individual can indirectly protect others by reducing transmission. The non-linear dynamics produced by this indirect protection (e.g., herd immunity) has played an important role in the success of vaccines against infectious diseases (e.g., eradication of smallpox [21]) and screening for sexually transmitted diseases (e.g., reduction of HIV by screening sex workers) [22]. It is particularly important to use transmission dynamic models (which inherently capture indirect effects), when examining the effectiveness and the cost-effectiveness of *C. difficile* prevention and control strategies, such as screening and vaccination, as an anticipated major benefit of such strategies is the reduction of *C. difficile* transmission within hospitals.

We conducted a systematic review of mathematical models of transmission dynamics of healthcare-associated *C. difficile* infection and colonization in order to provide an overview of current models and their predictions of the effectiveness of CDI control strategies. Specific aims were to describe and compare: 1) the models’ structure and assumptions about *C. difficile* natural history and transmission dynamics; and 2) *C. difficile* prevention and control strategies investigated by the models and their predictions of effectiveness.

## Methods

### Search strategy and selection criteria

We performed a systematic review of the literature, which we report according to PRISMA guidelines [23] (see S1 Table). The studies were eligible for inclusion in the systematic review if they included a mathematical model of transmission dynamics for hospital-acquired *C. difficile* infection or colonization. We searched MEDLINE (PubMed), EMBASE and Web of Science databases, with no restriction on the language of the articles, document type or year of publication. The last search was performed on February 3, 2016. We developed the search strategy by ensuring equivalent design and terminology in each database (with the use of Medical Subject Heading (MeSH) or Emtree terms used in PubMed and EMBASE respectively). The search strategy was divided into four groups of keywords concerning: #1) *Clostridium difficile*; AND #2) mathematical modeling; AND #3) hospitals and healthcare-associated infection; NOT #4) animals (see S1 Appendix for a detailed list of keywords). GG and MHG independently 1) identified eligible studies through review of titles and abstracts; 2) retrieved the full-text studies; and 3) assessed the eligibility of studies. To identify additional studies, we also reviewed the references of the selected articles.

### Data extraction for description of models

We extracted the data for the description of the models using an extraction form containing the following components: 1) model objectives and specifications; 2) natural history assumptions; 3) transmission pathways; 4) parameterization methods, calibration and uncertainty/sensitivity analysis; 5) *C. difficile* prevention and control strategies examined; and 6) model output/outcomes. Data extraction was performed independently by two reviewers (GG and MHG) using the predefined form. We contacted the authors of the studies to request further specific clarifications when information was lacking or unclear.

### Assessment of the quality of reporting

To our knowledge, no checklist or formal assessment tools are available to evaluate the quality of reporting for transmission-dynamic models. However, guidelines have been published concerning best modeling research practices. To assess the quality of the reporting of the models included in this systematic review, we developed a questionnaire-based grid, considering the recommendations from various modeling practice guidelines [24–41]. The questionnaire consisted of the following categories: 1) research question; 2) natural history representation and the transmission dynamics; 3) parameter estimates and data sources; 4) modeling approaches and mathematical methods; 5) outcomes of models; 6) uncertainty and sensitivity analyses; 7) validation and quality of documentation (see S2 Appendix for details). For all studies, every question was assessed in order to establish if the items defined in the question were fully/partially/not addressed by the study or if the items were not applicable. The quality of the reporting was assessed independently by two reviewers (GG and MHG).

### Representation of natural history and transmission dynamics

Natural history refers to the progression of the disease between health states in an individual over time. It can be represented schematically by a set of health state compartments linked by arrows indicating the possible transitions (progression/regression) between the health states. To compare the natural history representation of the models, we developed an epidemic model template that divides the natural history representations of *C. difficile* into six possible health states (S-E-A-I-R-D: Susceptible-Exposed-Asymptomatic-Infectious-Resistant-Deceased) (see S3 Appendix for details). For each model, the template allowed us to: 1) stratify every health state, according to modeled population characteristics; 2) clearly identify every health state transition defined in the natural history representation of the models; and 3) identify each transmission pathway.

### Effectiveness of *C. difficile* control strategies

For each primary health outcome defined, we extracted the effect of interventions and we reported its effectiveness. The outcomes of interest were the relative reduction in: 1) *C. difficile* colonization (incidence or ratio of colonized patients discharged to the colonized patients admitted); 2) CDI incidence; 3) incidence of recurrence of CDI; 4) CDI mortality; and 5) length of stay. We extracted the relative reduction in each outcome either directly from the studys’ text and tables, by using a graphical approach or by retrieving the simulation data from an author’s website [42]. In a second step, we categorized each of these interventions into one of the three groups according to their principal aim: 1) reducing the transmission; 2) reducing vulnerability to CDI; 3) reducing the risk of CDI recurrence or CDI mortality. Also, we reported the effectiveness of modeled bundles of interventions which can comprise any combination of the previous items.

## Results

### Study Selection

**Fig 1:**
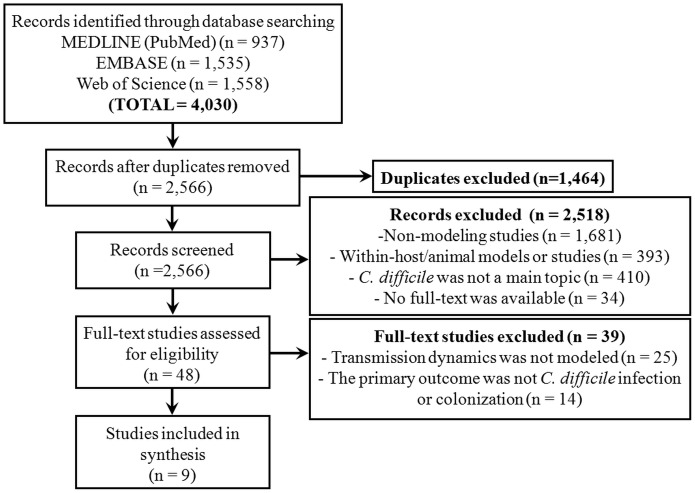
Flowchart of study selection.

We identified 2,566 records (articles and abstracts) after duplicates were removed. After screening of titles and abstract, 47 studies were selected for a full-text review (Fig 1). Nine studies met the inclusion criteria and were included in the systematic review. Within these nine studies, we identified six independent models (three studies were based on adaptations of previously published models). The studies were published between 2001 and 2015 and originate from three countries (the US, the UK, and Australia) (Table 1).

**Table 1:** Characteristics of studies included in the systematic review.

First author (year)	Starr, JM (2001) [43]	Starr, JM (2009) [44]	Lanzas, C (2011) [45]	Yakob, L (2013) [46]	Rubin, MA (2013) [47]	Lofgren, ET (2014) [48]	Yakob, L (2014) [49]	Lanzas, C (2014) [50]	Codella, J (2015) [51]
**GENERAL INFORMATION**									
Country	United Kingdom	United Kingdom	United States of America	Australia	United States of America	United States of America	Australia	United States of America	United States of America
Model adapted from another model included in this review	No	Yes. Adapted from Starr *et al.* (2001)	No	No	No	No	Yes. Adapted from Yakob *et al.* (2013)	Yes. Adapted from Lanzas *et al.* (2011)	No
Objectives	Increase knowledge of natural history	Evaluate the impact of interventions	Increase knowledge of natural history	Evaluate the impact of interventions	Evaluate the impact of interventions	Evaluate the impact of interventions	Evaluate the impact of interventions	Evaluate the impact of interventions	Evaluate the impact of interventions
Aggregate or individual-based	Not specified	Not specified	Aggregate and individual-based	Aggregate	Agent-based	Aggregate	Aggregate	Agent-based	Agent-based
**HOSPITAL SETTINGS**									
Settings	1 hospital ward for the elderly	2 hospital wards for the elderly	6 wards at a tertiary hospital	1 large hospital	11 hospital wards	1 intensive care unit (ICU)	1 large hospital	6 wards at a tertiary hospital	1 mid-sized hospital
Rooms/beds layout	4 rooms with 6 beds	Each ward has 4 separate rooms with 6 beds and 6 rooms with 1 bed	2 wards with 26 beds, 1 wards with 29 beds, and 3 wards with 30 beds	Not specified	9 acute care units (ACU) with 30 rooms, and 2 intensive care units (ICU) with 15 rooms (all private rooms)	12 rooms (1 bed per room)	Not specified	2 wards with 26 beds, 1 wards with 29 beds, and 3 wards with 30 beds	10 wards with 10 rooms (2 beds per room)
Number of beds	24 beds	60 beds	171 beds	1000 beds	300 beds	12 beds	1000 beds	171 beds	200 beds
**NATURAL HISTORY AND TRANSMISSION**									
Health states^a^	S-A-I-R	S-A-I-R	S-A-I-R	S-E-A-I	S-A-I	S-A-I	S-E-A-I	S-A-I-R	S-E-A-I-R-D
Hospital wards, rooms and beds layout taken into account in transmission (spatial heterogeneity)	Not specified	Yes. Transmission occurs between 1) patients in the same room, and 2) patients in different rooms	No. Transmission occurs homogeneously between patients at ward level	No. Transmission occurs homogeneously between patients at hospital level	Yes. Transmission occurs through HCWs within wards (nurses, doctors), across wards (doctors) and according to the patient location	No. Transmission occurs homogeneously between patients at ward level	No. Transmission occurs homogeneously between patients at hospital level	No. Transmission occurs homogeneously between patients at ward level	Yes. Transmission occurs between patients and HCW through movements within and between wards (for HCWs)
Transmission pathways	Patient-patient, environment-patient	Patient-patient, environment-patient	Patient-patient	Patient-patient	Environment-patient, HCW-patient	HCW-patient	Patient-patient	Patient-patient	Patient-patient, environment-patient, HCW/visitor-patient
*C. difficile* strain included in model	Single epidemic strain	Single epidemic strain	Not specified	Not specified	Toxigenic and non-toxigenic strains	Not specified	Not specified	Epidemic (ribotype 027) and other strains	Single epidemic strain
**MODEL UNCERTAINTY AND CALIBRATION**									
Sensitivity analyses carried out	Not specified	Yes	Yes	Yes	Yes	No	Yes	Yes	Yes
Model calibration	Not specified	Yes	Not specified	Not specified	Not specified	Not specified	Not specified	Not specified	Yes
**HEALTH OUTCOMES**									
CD colonization	—	—	Secondary cases generated by colonized or disease patients (R_0_)	Ratio of colonized discharged to colonized admitted	Cases per 10,000 patient-days	—	Ratio of colonized discharged to colonized admitted	Cases per 1,000 admissions	Cases per year and %^c^
CDI	Prevalence in the ward (number of cases)	Cases per year	Cases per 1,000 admissions	Cases per 1,000 hospital bed-days	Cases per 10,000 patient-days (actual and reported^b^)	Cases per year	Cases per 1,000 hospital bed-days	Cases per 1,000 admissions (CO and HO)	Cases per year and %^c^
CDI recurrence incidence	—	—	—	—	—	Cases per year	—	—	Cases per year and %^c^
CDI mortality	—	—	—	—	—	—	—	—	Cases per year and %^c^
Length of stay	—	—	—	—	—	—	—	—	Days
Measure of effect of interventions	Not applicable	Absolute reduction compared to the standard care scenario	Not applicable	Absolute reduction compared to the standard care scenario	Absolute reduction and percentage reduction compared to the standard care scenario	Absolute reduction compared to no intervention	Absolute reduction compared to the standard care scenario	Absolute reduction compared to no intervention, number needed to treat (NNT)	Absolute reduction compared to no intervention
**BASELINE EPIDEMIOLOGY**									
CD colonization	—	—	R_0_ from 0.52 to 1.99 (mean of 1.07 and median of 1.04)	7.5 (ratio of colonized patients discharged to colonized patients admitted)	152.6 cases per 10,000 patient-days	—	7.5 (ratio of colonized patients discharged to colonized patients admitted)	100 cases per 1,000 admissions	961 cases per year
CDI	Not specified	21.19 cases per year	17.85 cases per 1,000 admissions	2.8 cases per 1,000 hospital bed-days	14.3 cases per 10,000 patient-days (8.3 cases per 10,000 patient-days)^b^	Median(IQR): 0(0-1) cases per year, (0.81 cases per year)	2.8 cases per 1,000 hospital bed-days	14.5 cases (HO) per 1,000 admissions	601 cases per year
CDI recurrence incidence	—	—	—	—	—	Median(IQR): 2(0-6) cases per year, (4.35 cases per year)	—	—	142 cases per year
CDI mortality	—	—	—	—	—	—	—	—	122 cases per year
Length of stay	—	—	—	—	—	—	—	—	5.41 days

**CD:** *Clostridium difficile*; **CDI:** *Clostridium difficile* infection; **CO:** community-onset; **HCW:** healthcare worker; **HO:** hospital-onset; **IQR:** interquartile range.

^a^ Health states are categorized into four groups of models according to our epidemic model template that divides the natural history representations of *C. difficile* into six possible health states (S-E-A-I-R-D: Susceptible-Exposed-Asymptomatic-Infectious-Resistant-Deceased) (see S3 Appendix).

^b^ Reported CDI incidence is based on symptom recognition and positive results of *C. difficile* testing (enzyme immunoassay (EIA) test (sensitivity: 0.70; specificity: 0.97; turnaround time: 2 hours)).

^c^ Percentages of the whole hospital population for one year (the total number of patients admitted was not specified).

### Study & model characteristics

#### Settings

The hospital setting was very variable between the modeling studies. The models either included one or more hospital wards (n = 5) or an entire hospital (n = 4) (Table 1).

#### Natural history representation and transmission dynamics

**Fig 2:**
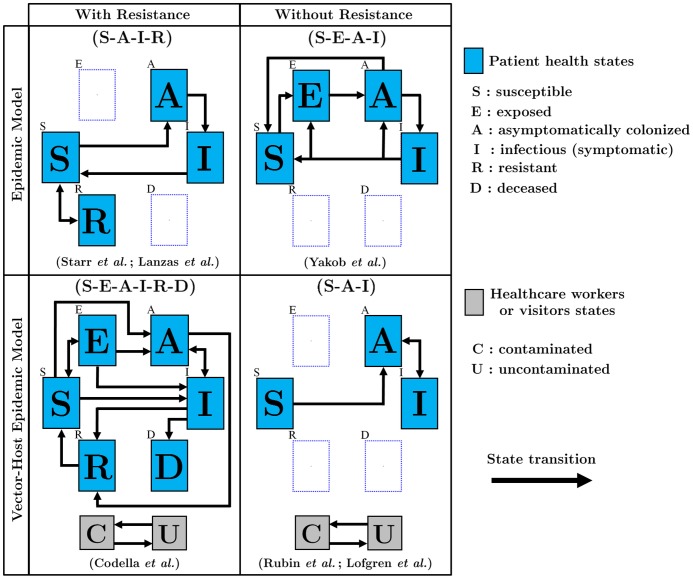
Natural history representations. Only the principal transitions are presented and stratifications were omitted from this figure for simplicity (e.g., heterogeneities related to the exposure to antibiotics). Patients can only be in one of the mutually exclusive health states at a given time (S-E-A-I-R-D: Susceptible-Exposed-Asymptomatic-Infectious-Resistant-Deceased). Two compartments (C and U) are included for interactions between patients and HCWs/visitors. See S3 Appendix for the full description of the natural history assumptions and transmission pathways included in each model.

Natural history representations were categorized into four groups of models (see Fig 2 and Table 1 and S3 Appendix for a detailed representation of the natural history of each model).

All models included Susceptible (S), Asymptomatically colonized (A) and Infectious symptomatic (I) health states. Two studies included at least two *C. difficile* strain types in their framework. Key differences in the natural history between models were whether or not they included latent Exposed (E) and/or Resistant/Immune (R) health states. The Exposed (E) health state refers to the acquisition of the pathogen through the ingestion of *C. difficile* spores and it represents a latent period of non-infectivity where the spores germinate into vegetative cells. For the five models that include resistance states (R), type and duration of resistance varied dramatically. Studies either assumed temporary resistance to colonization (related to the barrier protection provided by a healthy gut microbiota, n = 4) or natural immunity following clearance of *C. difficile* colonization/infection (n = 1).

#### Transmission

The majority of studies (n = 7) included transmission between patients (exposure to infectious patients (symptomatic and colonized)) (Table 1). However, a subgroup of studies (n = 5) also included indirect transmission pathways (exposure from a contaminated HCW/visitor/environment). Environment as a source of transmission was incorporated explicitly in four studies, three of which also included the layout of the healthcare services (wards, rooms, beds) when modeling transmission dynamics. For the other studies, the transmission was independent of the location of *C. difficile* infected patients in the hospital. All studies used stochastic mathematical methods for transmission dynamics. Two approaches were used to model transmission: 1) homogeneous mixing between individuals of the population (the force of infection is based upon the mass-action assumption) (n = 6); or 2) contact patterns/networks between individuals/agents (n = 3). In the first case, the force of infection is determined by: 1) the prevalence of asymptomatically colonized and/or symptomatic patients, and/or contaminated HCW; 2) the effective contact rates between infectious individuals (patients/HCW); and/or 3) a static background of environmental contamination. The second method relies on properties of agent-based modeling, which accounts for contacts between individuals or between individuals and the environment. This requires modeling: 1) patient spatial heterogeneities; 2) mobility of agents (patient, HCW/visitor); and/or 3) contact patterns or contact rates.

#### Parameter estimation/calibration and uncertainty analyses

Only two modeling studies clearly stated that they used calibration methods, identifying parameter values by fitting their models to empirical data (Table 1). For the other studies, most parameters were directly inferred from the literature. The majority of studies reported using parametric sensitivity analysis. However, no study reported examining structural uncertainties related to the natural history representation, transmission pathways or contact patterns/networks.

### Quality assessment of models reporting

Generally, the studies provided a clear description of: 1) research question; 2) model assumptions about natural history and transmission; and 3) modeling approaches and mathematical methods (S2 Appendix). On the other hand, the main limitations of study reporting were in the description of: 1) model outcomes, including their comparators (e.g., absence or partial reporting of the baseline values of health outcomes) or the denominators used (e.g., unknown time horizon or patient population considered); and 2) the uncertainty and sensitivity analyses. Indeed, for the great majority of studies, uncertainty analysis was limited to a few parameters without assessment of structural uncertainty. Finally, two studies reported model validation.

### Study results

Two main objectives were addressed in the modeling studies: 1) to increase the understanding of *C. difficile* natural history and transmission dynamics (n = 2); and 2) to assess the impact of interventions (n = 7) (Table 1).

#### Understanding *Clostridium difficile* natural history

Both studies examining *C. difficile* natural history and transmission dynamics suggested that transmission between hospitalized patients is a key determinant of CDI incidence. Starr *et al.* [43], highlighted that hospital outbreaks are driven by transmission between patients, and are not caused by an environmental reservoir. Lanzas *et al.* [45] suggested that antibiotics play a smaller role on hospital acquired CDI compared to *C. difficile* transmission from colonized patients and the length of stay of patients (which increases their risk of exposure to *C. difficile* during their hospitalization).

#### Interventions for the prevention and control of CDI

The predictions of models regarding the impacts of CDI prevention and control strategies are presented in Table 2. The measure of the impact of interventions is assessed using a comparison to current practices (n = 3) or no intervention (n = 2). Five studies examined targeted approaches to reduce *C. difficile* transmission within hospitals including: 1) reduction of average length of stay (n = 2); 2) screening on admission followed by isolation of colonized patients (n = 2); 3) hand hygiene with soap and water (n = 1); 4) enhancement of the environmental decontamination (n = 1); and 5) improvement of adherence to contact precautions in isolation rooms (n = 1). These interventions showed a reduction of CDI incidence ranging from 3% to 49%. Four studies analyzed interventions aimed at reducing patients’ vulnerability to CDI: 1) antimicrobial stewardship (n = 3); 2) administration of probiotics (n = 2); and 3) prophylactic use of fecal microbiota transplantation (FMT) (n = 2). The models predicted that these interventions could reduce the incidence of CDI by 5% to 43%. Two studies investigated interventions designed to reduce the risk of recurrence or mortality: 1) oral vancomycin as an initial treatment for CDI (n = 1); and 2) use of FMT for all patients with CDI (n = 1). For these interventions, the predicted reductions in CDI recurrence were 42% and 90% respectively. Moreover, the vancomycin therapy provided up to 69% reduction in CDI-related mortality and the greatest reduction in length of stay (22%). Finally, five studies evaluated the impact of bundles of interventions. These multi-pronged interventions strategies predicted the largest reductions in CDI incidence (between 14% and 84%) as well as in new colonizations by *C. difficile* (42% to 89%), CDI-related mortality (93%) and length of stay (22%).

**Table 2:** Relative reduction (%) in CDI outcomes resulting from interventions of CDI prevention and/or control.

	Relative reduction (%) in ***C. difficile*** health outcomes				
INTERVENTIONS OF CDI PREVENTION AND/OR CONTROL	CD colonization	CDI incidence	CDI recurrence incidence	CDI mortality	Length of stay
**INTERVENTIONS AIMED AT REDUCING THE TRANSMISSION**					
• **Reduction of the transmission rate**					
Starr *et al.*, (2009): (halving transmission from infected patients in the same room)	—	3	—	—	—
Starr *et al.*, (2009): (halving transmission from infected patients in other rooms)	—	9	—	—	—
Starr *et al.*, (2009): (halving all transmission sources (from all infected patients within same/other rooms and from the environmental contamination))	—	15	—	—	—
Yakob *et al., (2013,2014):* (improved hand hygiene and sanitation, (no transmission))	77	49	—	—	—
• **Reduction of patients length of stay**					
Yakob *et al.*, (2013,2014): (length of stay reduced from 6 days to 3 days)	66	38	—	—	—
• **Screening on admission and isolation of colonized patients**					
Yakob *et al.*, (2013): (screening with a sensitivity of 100%)	11	7	—	—	—
Lanzas *et al.*, (2014): (PCR with sensitivity of 90% and turnaround time of 1 day)	41–43	14–25	—	—	—
Lanzas *et al.*, (2014): (PCR with sensitivity of 95% and turnaround time of 0.5 day)	52	25	—	—	—
• **Improved use of soap and water for hand hygiene**					
Codella *et al.*, (2015): (mean adherence of 48%)	10	20	18	19	5
• **Improved environmental decontamination (routine (24h) bleach of patient rooms)**					
Codella *et al.*, (2015): (100% adherence)	22	43	42	43	9
• **Improved adherence/performing contact precautions in isolation rooms**					
Codella *et al.*, (2015): (mean adherence of 62%)	12	23	24	24	5
**INTERVENTIONS AIMED AT REDUCING VULNERABILITY TO CDI**					
• **Reduction of the rate of becoming vulnerable to CDI**					
Starr *et al.*, (2009): (halving the rates of resistant patients becoming susceptible)	—	43	—	—	—
Yakob *et al.*, (2013,2014): (reduction of antibiotic prescription (0 per day))	44	13	—	—	—
• **Prophylactic use of FMT: (proportion of patients treated from 20% to 100%)**					
Lofgren *et al.*, (2014): (among patients exposed to high risk antimicrobials)	—	5–28	0–9	—	—
Lofgren *et al.*, (2014): (among patients exposed to high risk antimicrobials and PPI)	—	7–27	0-1	—	—
• **Use of probiotics/FMT in susceptible patients to expedite gut microbiota recovery**					
Yakob *et al.*, (2013,2014): (gut microbiota recovery time varied from 90 days to 10 days)	26	11	—	—	—
**INTERVENTIONS AIMED AT REDUCING THE RISK OF CDI RECURRENCE OR CDI MORTALITY**					
• **Use of FMT after CDI: (proportion of patients treated from 20% to 100%)**					
Lofgren *et al.*, (2014): (discharged patients (cleared of CDI or in process of recovering))	—	5–16	28–90	—	—
• **Vancomycin as initial treatment**					
Codella *et al.*, (2015): (oral vancomycin (2g/day), CDI diagnostic accuracy of 100%)	15	29	42	69	22
**BUNDLES OF INTERVENTIONS**					
**Starr *et al.*, (2009):** 1 bundle of 2 interventions					
• Halving all rates of becoming susceptible and halving all transmission rates	—	54	—	—	—
**Rubin *et al.*, (2013):** 2 types of bundles (typical/optimal) including 6 interventions each: 1) improved adherence with hand hygiene; 2) improved hand hygiene with soap and water during contact with CDI patients; 3) improved contact precautions during contact with CDI patients; 4) improved environmental decontamination; 5) aggressive/early testing; and 6) empiric isolation and treatment of suspected cases.					
• **Typical bundle of interventions:** improvement over the base-case values expected from a hospital focusing on improving adherence practices.					
∘ Model predictions	61–67	68–74	—	—	—
∘ Reported CDI incidence (positive results of *C. difficile* tests (EIA test))	—	44–57	—	—	—
• **Optimal bundle of interventions:** maximum effects anticipated from enhanced adherence practices and an aggressive campaign to reduce the pathogen transmission.					
∘ Model predictions	74–77	80–83	—	—	—
∘ Reported CDI incidence (positive results of *C. difficile* tests (EIA test))	—	57–63	—	—	—
**Lofgren *et al.*, (2014):** 1 bundle of 2 interventions					
• Prophylactic use of FMT among patients exposed to high risk antimicrobials and PPI and use of FMT after CDI. (The proportion of patients treated is from 20% to 100%.)	—	14–25	16–89	—	—
**Yakob *et al.*, (2014):** 6 bundles of 2 interventions					
• Reduction of antibiotic prescriptions and use of probiotics	69	30	—	—	—
• Reduction of antibiotic prescriptions and reduction of length of stay	75	59	—	—	—
• Use of probiotics and reduction of length of stay	65	64	—	—	—
• Improved hand hygiene/sanitation and reduction of antibiotic prescriptions	89	65	—	—	—
• Improved hand hygiene/sanitation and use of probiotics	89	66	—	—	—
• Improved hand hygiene/sanitation and reduction of length of stay	86	68	—	—	—
**Codella *et al.*, (2015):** 1 bundle of 4 interventions					
• Combination of 4 interventions: 1) vancomycin as initial treatment; 2) improved adherence when performing contact precautions in isolation rooms; 3) improved hand hygiene with soap and water; and 4) improved environmental decontamination (routine bleach of patient rooms).	42	84	86	93	22

**CD:** *Clostridium difficile*; **CDI:** *Clostridium difficile* infection; **EIA:** enzyme immunoassay; **FMT:** fecal microbiota transplantation; **PCR:** polymerase chain reaction; **PPI:** proton-pumps inhibitors.

## Discussion

To our knowledge, this is the first systematic review of transmission-dynamic mathematical models of *C. difficile* infection and colonization in healthcare settings. Only six distinct models were identified in the literature, despite: 1) the important health and economic burden of *C. difficile* infections on healthcare systems; and 2) the extensive use of such models to predict effectiveness and cost-effectiveness of prevention and control strategies for other infectious diseases to inform policy decisions [19, 52–54]. The models differed substantially in their natural history representations, healthcare settings modeled, outcome measures and interventions examined, which made it impossible to draw specific conclusions about optimal CDI prevention and control strategies. However, generally, the models suggested that individual or groups of interventions aimed at reducing *C. difficile* transmission within healthcare settings were more effective than interventions aimed at reducing CDI vulnerability, CDI recurrence or CDI mortality. Finally, the more recent models tended to be more complex, by integrating greater patient heterogeneity (e.g., local contamination levels depending on the spatial location of patients in the hospital or differential risks of colonization/CDI by level of antibiotic exposure), and more elaborate contact patterns/networks and interventions.

According to good modeling practice guidelines [25–27, 32, 35], model validation should include a between-model corroboration (convergent validity) and, when possible, external and predictive validation. Our systematic review shows that there is important variability in the predictions of the effectiveness of CDI control and prevention strategies which are due to the significant heterogeneity of interventions assessed through various natural history representation structures. *C. difficile* transmission-dynamic models examined three main categories of interventions for which we can consider external validity: 1) measures aimed at cutting transmission paths (mostly by environmental decontamination); 2) practices to prevent colonization/infection (e.g., antimicrobial stewardship programs); and 3) bundles of interventions. Firstly, the predicted effectiveness of interventions aimed at reducing CDI incidence through transmission control were generally in line with results from epidemiological studies. For example, these studies have shown that enhancing environmental decontamination (daily/terminal cleaning of hospital rooms) can reduce CDI incidence by 38–85% [55–61], which is similar to the model predictions of the effectiveness of environmental decontamination (43% compared to no intervention [51]) and hand hygiene and sanitation (49% compared to standard care [46]). Secondly, only two models investigated the impact of antimicrobial stewardship as an intervention. When examined, antimicrobial stewardship practices were indirectly incorporated into the models through an overall rate of antibiotic prescription without taking into account the differential risks of CDI according to antimicrobial classes. Both models predicted moderate (43% [44]) or little impact (11% [46]) of antibiotic use on CDI incidence, which contrasts with the moderate to high empirical effectiveness of antimicrobial stewardship programs at reducing CDI incidence (by 36–79% [14, 15, 62–68]). Thirdly, the models predicted that the effectiveness of bundles of interventions in preventing CDI incidence is 15–84%, which is similar to reductions reported by observational and intervention studies (31–71%) [69–73]. However, given the wide intervals and variability in the interventions included in the bundles, it is very difficult to conclude that the models are accurately predicting empirical outcomes.

There are three main uncertainties about the natural history, transmission and overall epidemiology of *C. difficile* in healthcare settings, which make it very difficult to model CDI control and prevention strategies. Firstly, the role of asymptomatically colonized patients in the transmission dynamics of *C. difficile* remains unclear (e.g., their contributions to new infections/colonizations and their period of incubation have not been precisely measured). However, evidence strongly suggests that asymptomatically colonized patients play an important role in *C. difficile* transmission within healthcare settings. Asymptomatically colonized patients are a source of importation of toxigenic *C. difficile* strains [74] and form a large reservoir for horizontal transmission [5, 75]. Furthermore, routine screening and isolation/cohorting of asymptomatic patients has been shown to be effective in decreasing CDI incidence [76]. Secondly, empirical data on the role/contribution of HCWs on overall transmission of *C. difficile*, especially due to their contact patterns/networks with patients and others HCWs, are lacking. The third key knowledge gap is the level of immunity/resistance to *C. difficile* colonization and development of disease. The basic resistance to *C. difficile* relies primarily on the presence of commensal bacteria, which confer protection against colonization. As depicted in the majority of *C. difficile* models, the use of antibiotics can disrupt these protective bacterial populations, which can lead to colonization of the intestinal tract by *C. difficile* and a higher risk of CDI. However, other forms of immunity/resistance have not been explored by the models, such as, for example, the effect of an anamnestic immune response after clearance of infection from a first episode on the risk of CDI recurrences.

We identified four modeling gaps. Firstly, antimicrobial stewardship programs modeled should include more mechanistic elements in order to examine the impact of various stewardship strategies, which would be informative for decision makers. In particular, future models could include differential risks of CDI associated with antibiotic classes or could examine how CDI health outcomes are affected by treatment duration and antimicrobial consumption. Secondly, none of the transmission-dynamic models have examined the cost-effectiveness of CDI interventions, although such analyses are currently a crucial element in policy decisions. Thirdly, structural uncertainty analysis should be included and reported more systematically, and the model should be cross-validated with observational studies. The variability in model predictions of effectiveness found in this review could be explained by the wide variety of model structures and interventions examined. Examining the impact of structural assumptions would provide insights on which assumptions have the greatest impact on model predictions, and allow better assessment of the validity of the models. For example, it would be important to examine whether specific assumptions about transmission, due to the focus on transmission dynamics, could explain why interventions aimed at reducing transmission were more effective than antibiotic stewardship (which does not seem to be consistent with the results from epidemiological studies). Fourthly, the emergence of more virulent strains has changed the epidemiology of *C. difficile* during the last decade. However, although significantly higher risk of colonization/CDI/recurrence has been linked with some specific hypervirulent *C. difficile* strains (e.g., BI/NAP1/027 [4, 77] or BK/NAP7/078 [78]), this has not systematically been included in published *C. difficile* transmission-dynamic models. Including strain-specific transition rates would allow models to capture the differential risk of CDI morbidity (e.g., severe infection, complications, ICU admission, treatment failure due to antimicrobial resistance) and mortality between historic and hypervirulent strains [79–81]. Finally, the review highlights key features that modelers should consider including in their models to increase their usefulness and validity. Models should preferably be agent-based, allowing an easier integration of environmental heterogeneity of the healthcare settings and consequently of the transmission pathways between patients and other agents such as healthcare workers (e.g., Rubin *et al.* [47] and Codella *et al.* [51] use such models). More importantly, models should be calibrated to epidemiological data and model fit should be shown/described.

## Conclusions

Mathematical models of transmission dynamics of *Clostridium difficile* in healthcare settings are scarce considering the significant burden generated by this pathogen on healthcare systems. Current models vary substantially in their natural history assumptions, outcome measures presented and interventions examined, which lead to an unclear picture of the optimal intervention strategies to control and prevent *C. difficile* infections. Future modeling efforts should pay specific attention to calibration, structural uncertainties, cost-effectiveness and good transparency practices.

## Supporting Information

S1 Table PRISMA checklist. (PDF)

S1 Appendix Search strategy. (PDF)

S2 Appendix Assessment of the quality of reporting of the transmission dynamics mathematical models. (PDF)

S3 Appendix Analysis of the natural history representations and transmission dynamics. (PDF)
