# Supplementary material for: Mathematical Modeling of the Transmission Dynamics of Clostridium difficile Infection and Colonization in Healthcare Settings: A Systematic Review
Source: PLoS One. 2016 Sep 30;11(9):e0163880. doi: 10.1371/journal.pone.0163880 (PMC5045168; doi:10.1371/journal.pone.0163880)
Supplement: S1 Appendix — (PDF) [file pone.0163880.s002.pdf]

S1 Appendix. Search strategy

Reporting of the search strategy

Host platform and databases searched:

1. **PubMed sources:** MEDLINE
2. **EMBASE sources:** EMBASE and MEDLINE
3. **WEB OF SCIENCE sources:** all databases (Web of Science Core Collection (1900-present); Current Contents Connect (1998-present); KCI-Korean Journal Database (1980-present); MEDLINE (1950-present); Russian Science Citation Index (2005-present); SciELO Citation Index (1997-present))

Date last search was run: February 3, 2016.

Years covered by search: All years available in each database were included in the search.

Language restriction: No language restriction.

Document restriction: No document types restriction.

Summary of the search strategy: The search strategy was developed to ensure a similar design/terminology (with the inclusion of the controlled vocabulary: Medical Subject Heading (MeSH) or Emtree terms used respectively in PubMed and EMBASE) across the three host platforms of databases searched. The search strategy is divided into four groups of keywords:

#1: Clostridium difficile.
#2: Mathematical models.
#3: Hospitals and healthcare-associated infections.
#4: Animals.

The four groups were combined with the following logic: **#1 AND #2 AND #3 NOT #4**.

MEDLINE (PUBMED) (937 results)

- **#1-** (”Clostridium difficile”[Title/Abstract] OR ”C. diff.”[Title/Abstract] OR ”C. difficile”[Title/Abstract] OR ”Clostridium difficile”[Mesh] OR ”Enterocolitis, Pseudomembranous”[Mesh])

- **#2-** (mathematic*[Title/Abstract] OR model*[Title/Abstract] OR simulation*[Title/Abstract] OR dynamic*[Title/Abstract] OR determinist*[Title/Abstract] OR stochastic[Title/Abstract] OR ”agent-based”[Title/Abstract] OR decision*[Title/Abstract] OR ”Monte Carlo”[Title/Abstract] OR compartmental[Title/Abstract] OR bayesian[Title/Abstract] OR Markov[Title/Abstract] OR economic*[Title/Abstract] OR cost[Title/Abstract] OR ”cost-effectiveness”[Title/Abstract] OR ”cost-efficacy”[Title/Abstract] OR ”cost-efficiency”[Title/Abstract] OR ”Models, Theoretical”[Mesh] OR ”Computer Simulation”[Mesh] OR ”Models, Economic”[Mesh])

- **#3-** (hospital*[Title/Abstract] OR admission*[Title/Abstract] OR discharge*[Title/Abstract] OR ”intensive care”[Title/Abstract] OR ”acute care”[Title/Abstract] OR ”care unit”[Title/Abstract] OR surgery[Title/Abstract] OR ward*[Title/Abstract] OR room*[Title/Abstract] OR bed[Title/Abstract] OR beds[Title/Abstract] OR nosocomial[Title/Abstract] OR patient*[Title/Abstract] OR inpatient*[Title/Abstract] OR outpatient*[Title/Abstract] OR healthcare[Title/Abstract] OR ”health care”[Title/Abstract] OR ”Health Facilities”[Mesh] OR ”Cross Infection”[Mesh] OR ”Patients”[Mesh])

- **#4-** (”animals, domestic”[Mesh] OR ”animals, laboratory”[Mesh] OR ”Models, animal”[Mesh])

#1 AND #2 AND #3 NOT #4 --- (n = 937)

COMPLETE STRATEGY (Copy and paste):

(”Clostridium difficile”[Title/Abstract] OR ”C. diff.”[Title/Abstract] OR ”C. difficile”[Title/Abstract] OR ”Clostridium difficile”[Mesh] OR ”Enterocolitis, Pseudomembranous”[Mesh]) AND (mathematic*[Title/Abstract] OR model*[Title/Abstract] OR simulation*[Title/Abstract] OR dynamic*[Title/Abstract] OR determinist*[Title/Abstract] OR stochastic[Title/Abstract] OR ”agent-based”[Title/Abstract] OR decision*[Title/Abstract] OR ”Monte Carlo”[Title/Abstract] OR compartmental[Title/Abstract] OR bayesian[Title/Abstract] OR Markov[Title/Abstract] OR economic*[Title/Abstract] OR cost[Title/Abstract] OR ”cost-effectiveness”[Title/Abstract] OR ”cost-efficacy”[Title/Abstract] OR ”cost-efficiency”[Title/Abstract] OR ”Models, Theoretical”[Mesh] OR ”Computer Simulation”[Mesh] OR ”Models, Economic”[Mesh]) AND (hospital*[Title/Abstract] OR admission*[Title/Abstract] OR discharge*[Title/Abstract] OR ”intensive care”[Title/Abstract] OR ”acute care”[Title/Abstract] OR ”care unit”[Title/Abstract] OR surgery[Title/Abstract] OR ward*[Title/Abstract] OR room*[Title/Abstract] OR bed[Title/Abstract] OR beds[Title/Abstract] OR nosocomial[Title/Abstract] OR patient*[Title/Abstract] OR inpatient*[Title/Abstract] OR outpatient*[Title/Abstract] OR healthcare[Title/Abstract] OR ”health care”[Title/Abstract] OR ”Health Facilities”[Mesh] OR ”Cross Infection”[Mesh] OR ”Patients”[Mesh]) NOT (”animals, domestic”[Mesh] OR ”animals, laboratory”[Mesh] OR ”Models, animal”[Mesh])

EMBASE (1,535 results)

- **#1-** (”Clostridium difficile”:ab,ti OR ”C. diff.”:ab,ti OR ”C. difficile”:ab,ti OR ”Clostridium difficile infection”/exp OR ’Peptoclostridium difficile’/exp OR ’pseudomembranous colitis’/exp)

- **#2-** (mathematic*:ab,ti OR model*:ab,ti OR simulation*:ab,ti OR dynamic*:ab,ti OR determinist*:ab,ti OR stochastic:ab,ti OR ”agent-based”:ab,ti OR decision*:ab,ti OR compartmental:ab,ti OR bayesian:ab,ti OR Markov:ab,ti OR ”Monte Carlo”:ab,ti OR economic*:ab,ti OR cost:ab,ti OR ”cost effectiveness”:ab,ti OR ”cost efficacy”:ab,ti OR ”cost efficiency”:ab,ti OR ”computer model”/exp OR ”mathematical model”/exp OR ”economic evaluation”/exp)

- **#3-** (hospital*:ab,ti OR admission*:ab,ti OR discharge*:ab,ti OR ”intensive care”:ab,ti OR ”acute care”:ab,ti OR ”care unit”:ab,ti OR surgery:ab,ti OR ward*:ab,ti OR room*:ab,ti OR bed:ab,ti OR beds:ab,ti OR nosocomial:ab,ti OR patient*:ab,ti OR inpatient*:ab,ti OR outpatient*:ab,ti OR healthcare:ab,ti OR ”health care”:ab,ti OR ”health care facility”/exp OR ”nosocomial infection”/exp OR ”patient”/exp)

- **#4-** (”Nonhuman”/exp OR ”animal experiment” OR ”experimental animal”/exp OR ”animal model”/exp)

#1 AND #2 AND #3 NOT #4 --- (n = 1,535)

COMPLETE STRATEGY (Copy and paste):

(”Clostridium difficile”:ab,ti OR ”C. diff.”:ab,ti OR ”C. difficile”:ab,ti OR ”Clostridium difficile infection”/exp OR ’Peptoclostridium difficile’/exp OR ’pseudomembranous colitis’/exp) AND (mathematic*:ab,ti OR model*:ab,ti OR simulation*:ab,ti OR dynamic*:ab,ti OR determinist*:ab,ti OR stochastic:ab,ti OR ”agent-based”:ab,ti OR decision*:ab,ti OR compartmental:ab,ti OR bayesian:ab,ti OR Markov:ab,ti OR ”Monte Carlo”:ab,ti OR economic*:ab,ti OR cost:ab,ti OR ”cost effectiveness”:ab,ti OR ”cost efficacy”:ab,ti OR ”cost efficiency”:ab,ti OR ”computer model”/exp OR ”mathematical model”/exp OR ”economic evaluation”/exp) AND (hospital*:ab,ti OR admission*:ab,ti OR discharge*:ab,ti OR ”intensive care”:ab,ti OR ”acute care”:ab,ti OR ”care unit”:ab,ti OR surgery:ab,ti OR ward*:ab,ti OR room*:ab,ti OR bed:ab,ti OR beds:ab,ti OR nosocomial:ab,ti OR patient*:ab,ti OR inpatient*:ab,ti OR outpatient*:ab,ti OR healthcare:ab,ti OR ”health care”:ab,ti OR ”health care facility”/exp OR ”nosocomial infection”/exp OR ”patient”/exp) NOT (”Nonhuman”/exp OR ”animal experiment” OR ”experimental animal”/exp OR ”animal model”/exp)

WEB OF SCIENCE (1,558 results)

- **#1-** TS=(”Clostridium difficile” OR ”C. diff.” OR ”C. difficile”)
- **#2-** TS=(mathematic* OR model* OR simulation* OR dynamic* OR determinist* OR stochastic OR agent-based OR decision* OR ”Monte Carlo” OR compartmental OR bayesian OR Markov OR economic* OR cost* OR ”cost-effectiveness” OR ”cost-efficacy” OR ”cost-efficiency”)
- **#3-** TS=(hospital* OR *admission* OR discharge* OR ”intensive care” OR ”acute care” OR ”care unit” OR surgery OR ward* OR room* OR bed OR beds OR nosocomial OR patient* OR inpatient* OR outpatient* OR health* OR ”health care”)
- **#4-** TS=(animal*)

#1 AND #2 AND #3 NOT #4 --- (n = 1,558)

COMPLETE STRATEGY (Copy and paste):

TS=(”Clostridium difficile” OR ”C. diff.” OR ”C. difficile”) AND TS=(mathematic* OR model* OR simulation* OR dynamic* OR determinist* OR stochastic OR agent-based OR decision* OR ”Monte Carlo” OR compartmental OR bayesian OR Markov OR economic* OR cost* OR ”cost-effectiveness” OR ”cost-efficacy” OR ”cost-efficiency”) AND TS=(hospital* OR *admission* OR discharge* OR ”intensive care” OR ”acute care” OR ”care unit” OR surgery OR ward* OR room* OR bed OR beds OR nosocomial OR patient* OR inpatient* OR outpatient* OR health* OR ”health care”) NOT TS=(animal*)
