# Supplementary material for: Mathematical Modeling of the Transmission Dynamics of Clostridium difficile Infection and Colonization in Healthcare Settings: A Systematic Review
Source: PLoS One. 2016 Sep 30;11(9):e0163880. doi: 10.1371/journal.pone.0163880 (PMC5045168; doi:10.1371/journal.pone.0163880)
Supplement: S2 Appendix — (PDF) [file pone.0163880.s003.pdf]

S2 Appendix. Assessment of the quality of reporting of the transmission-dynamic mathematical models

We have developed a questionnaire-based grid to assess the quality of reporting of the transmission dynamics models of *Clostridium difficile* in healthcare settings. Our grid is primarily intended to assess within each study, the abilities of authors to efficiently describe their model and to report the ensuing findings appropriately. We based our criteria in accordance with various existing modeling practice guidelines to inform policy decisions. These guidelines come from various fields of expertise such as modeling in environmental regulation [1, 2] and healthcare modeling to inform medical decisions and the management of healthcare resources [3, 4, 5, 6, 7, 8], which specifically included modeling of the transmission dynamics [9] and model-based economic evaluations (decision-analytic models) [10, 11, 12, 13, 14, 15].

Questionnaire-based grid to assess the quality of reporting of dynamic models

The assessment of the quality of reporting consists into 35 questions which are divided in seven sections containing each five questions. The sections are related to the following elements:

1) research question;
2) natural history representation and the transmission dynamics;
3) parameter estimates and data sources;
4) modeling approaches and mathematical methods;
5) model outcomes;
6) uncertainty analyses and sensitivity analyses;
7) validation and the quality of documentation.

Rating scale definition for the quality of reporting criteria

For the evaluation of each question, we used a four-point rating scale defined as follows:

NOT APPLICABLE (N/A): The question does not apply to the study.
LOW (L): The question has not been addressed by the study.
MEDIUM (M): The question has been partially addressed by the study.
HIGH (H): The question has been fully addressed by the study.

Description of the quality of reporting criteria

Below, we provide a brief explanation about the seven sections used in our assessment of the quality of reporting. The questionnaire can be applied to evaluate the quality of reporting of a general dynamic model applied to infectious disease. The seven different sections, used in our assessment, are defined as follows:

1. **Research question:** the study must determine clearly an objective or a problem being addressed [11, 12, 2, 16, 7, 14, 13, 8]. In addition, the study must provide information on the target population [11, 7, 14, 13, 15, 8] and the healthcare settings being modeled [11, 7, 14, 15]. A time frame should also be provided [13, 14] in order to contextualize the study within the previous research or to understand the time interval represented by the model. Finally, the study should define the outcomes of interest and indicate their relevance to the research question being addressed [3, 11, 12, 14, 13, 7, 15, 8].

2. **Natural history representation and the transmission dynamic:** the natural history of the disease must be fully specified [3, 11, 12] by using a description of each health state and by showing each transition between them. Moreover, the study should provide a rationale [3, 11, 12, 14, 13, 8] about the structure chosen to represent the natural history. The study must define the type of unit of representation (aggregate population or individuals) which is used to model the target population [7, 8] and should report the inclusion of population heterogeneities [3, 11, 14, 13, 8]. The modeling of heterogeneities can be done by using additional stratifications in the natural history representation of the disease or by the use of an agent-based model which can track individuals with their specific characteristics. Finally, the transmission/exposure pathways (direct/indirect) of the pathogen that are included in the natural history must be clearly reported or be easily identifiable either by a textual description or by conceptual/logic diagrams.

3. **Parameter estimates and data sources:** every parameter used in the model should be reported (by a point estimate or by a distribution) [9, 14, 13, 8] with their appropriate units. The units should be consistent with dimensions of physical quantities that these parameters are intended to represent (e.g., a rate should have a dimension of 1/(unit of time) or a probability should be dimensionless). Each study should describe the methodologies and data sources used to identify the parameter estimates. These methods can include: expert elicitation [3, 11, 12, 4, 13]; literature reviews [3, 11, 4]; data analyses [11, 12, 2, 4, 13, 8]; calibration methods [2, 4]. All sources of data must be reported using citations.

4. **Modeling approaches and mathematical methods:** the study should provide justification for the modeling approach (e.g., aggregate model vs. individual/agent-based model, deterministic vs. stochastic) [11, 12, 4, 5, 14, 13, 15, 8] and should report the mathematical methods (e.g., set of differential equations, Markov chains, Monte Carlo methods, logic rules) used for the transmission dynamics (i.e., progression of the disease over time) [9]. All the interactions modeled between the target population and other model components must be reported [8] by specifying where they occur in the model architecture. For the main findings of the study, the time horizon, the cycle length and the number of simulations done should be reported [3, 11, 12, 7, 14, 13, 8]. Also, the information about the simulation software and programming language should be reported [11, 16, 4].

5. **Model outcomes:** the study must provide a clear definition for each outcome of interest [14] and should report clearly their baseline values (before any intervention). For the principal findings of the study, the outcomes must be reported numerically or should be presented in such a way that allows data extraction. Furthermore, the units used for the outcome measurements should be clearly defined in such a way that leaves no space for ambiguity or for misinterpretation (i.e., by using clear definitions of numerators/denominators and time horizon). Finally, the study should report a graphical representation of the transmission dynamics over time (e.g., incidence and/or prevalence of infection/disease) [9].

6. **Uncertainty analyses and sensitivity analyses:** the study should assess and then reported any kind of uncertainty analysis (to assess the effect of a lack of knowledge or the inherent variability in the model) or sensitivity analysis (a measure of the effect of a change in model outputs by varying the input values) that were conducted. The different types of uncertainty include: stochastic uncertainty (variability in outcomes) [17, 5, 13], parametric uncertainty (variability in the model inputs (parameters-specific)) [11, 17, 12, 16, 4, 5, 14, 13, 8], structural uncertainty (variability in the model choice of representation for the biological properties of the disease (natural history)) [11, 12, 16, 5, 9, 7, 14, 13, 8] and the calibration uncertainty (variability in the parameter-space, methods and calibrated parameters) [1, 17, 4, 5, 8]. The study should report any sensitivity analysis [8, 11, 12, 2, 16, 13, 3, 4, 5, 17] that has been carried out (deterministic sensitivity analysis (DSA), probabilistic sensitivity analysis (PSA) or both).

7. **Validation and the quality of documentation:** the study should report if the model has been validated (face/internal/cross/external/predictive- validity) [10, 3, 11, 17, 12, 4, 6, 13, 15, 8] and should acknowledge the limitations and the strengths of the model. [11, 2, 16, 14, 8]. The study should provide complementary information (non-technical or technical) in the forms of appendices, supplementary files or should mention the availability of complementary information on request [16, 6, 15, 8]. Some graphical descriptions of model concepts should be given [8]. Finally, the study should not contain apparent errors, inconsistencies or missing information.

Results

The nine studies [18, 19, 20, 21, 22, 23, 24, 25, 26] included in our review were assessed independently by two reviewers for their quality of reporting and we reported it in detail in **Table S2.1**. The study of Starr *et al.* (2001) [18] was evaluated differently because the study consisted in an ”update article” for which the primary intent was to show the usefulness of dynamic modeling for *C. difficile* infection in order to study the sources of transmission in a hospital setting. In this case, the authors emphasize more on the rationales related to the use of a model of transmission dynamics rather than to report exhaustively their model in all details. Consequently, many questions were not applicable (N/A) for this study.

The percentage of a perfect agreement between the two reviewers reached 76% overall (excluding the items not applicable (N/A)). The few discrepancies between the two reviewer scores that were different by more than one adjacent point were assessed again and, then resolved by consensus or by using a third reviewer. The discrepancies were reported in **Table S2.1** using intermediary scales (see **Table S2.2**) which represents the scores given by the two reviewers.

Tables S2.1: Rating of the quality of the reporting for the mathematical models of transmission dynamics of *Clostridium difficile* infection and colonization in healthcare settings.

		First author, (year)								
**Q#**	**Question of review**	**Starr (2001) [18]**	**Starr (2009) [19]**	**Lanzas (2011) [20]**	**Yakob (2013) [21]**	**Rubin (2013) [22]**	**Lofgren (2014) [23]**	**Yakob (2014) [24]**	**Lanzas (2014) [25]**	**Codella (2015) [26]**
	**1-RESEARCH QUESTION**									
**1**	Is there a clear statement of the problem/objective?	M	M	H	H	H	H	H	H	H
2	Has a target population been identified?	H	H	H	H	H	H	H	H	H
**3**	Has a setting or location been defined?	H	H	H	M-H	H	H	M-H	H	H
4	Has a time frame been defined?	H	H	H	H	H	H	H	H	M
**5**	Are the health outcomes relevant to the research question?	M-H	H	H	H	H	H	H	H	H
	**2-NATURAL HISTORY REPRESENTATION AND THE TRANSMISSION DYNAMICS**									
6	Has natural history been clearly defined (health states and transitions)?	M	M-H	H	H	M	H	H	L-M	M
**7**	Is there a discussion/rationale regarding the structure of the natural history?	H	M-H	H	H	M-H	H	H	M-H	L-M
8	Have the units of representation (for the target population modeled) been clearly defined (aggregate/cohort vs. individuals)?	N/A	L-M	M-H	H	H	H	H	H	H
**9**	Are population heterogeneities reported/considered and are justifications provided?	N/A	H	H	H	H	H	H	M-H	M-H
**10**	Has each transmission pathway been clearly defined?	N/A	H	H	M-H	M-H	H	H	M-H	M-H
	**3-PARAMETER ESTIMATES AND DATA SOURCES**									
**11**	Are every parameters fully defined with the appropriate units?	N/A	M	H	M-H	M-H	M-H	M-H	M-H	L-M
**12** **12**	Are parameter estimates obtained from experts elicitation reported with methodologies?	N/A	N/A	N/A	N/A	M-H	N/A	N/A	N/A	M
**13**	Are parameter estimates obtained from literature review reported with methodologies and data sources?	N/A	N/A	M	M	M	M	M	M	M
**14**	Are parameter estimates obtained from data analysis reported with methodologies and data sources?	N/A	H	M-H	N/A	M-H	M	N/A	M-H	M
**15**	Are parameter estimates obtained from calibration reported with methodologies and data sources?	N/A	M-H	N/A	N/A	N/A	N/A	N/A	N/A	M-H
	**4-MODELING APPROACHES AND MATHEMATICAL METHODS**									
**16**	Has a justification been given for the modeling approach?	H	H	H	H	H	H	H	M-H	H
**17**	Have the mathematical methods used for the transmission dynamics been reported and justified?	M-H	H	M-H	H	H	H	H	L-M	H
**18**	Have every interactions (modeled within the target population) been reported?	N/A	M-H	H	H	M-H	H	H	L-M	H
**19**	For the main findings of the study, are the time horizon, the cycle length and number of simulation runs done reported?	N/A	H	M	H	M-H	H	M	L-M	M
**20**	Are there information concerning the simulation software?	N/A	L	L	L	H	H	L	H	H

Tables S2.1--(continued from previous page): Rating of the quality of the reporting for the mathematical models of transmission dynamics of *Clostridium difficile* infection and colonization in healthcare settings.

		First author, (year)								
**Q#**	**Question of review**	**Starr (2001) [18]**	**Starr (2009) [19]**	**Lanzas (2011) [20]**	**Yakob (2013) [21]**	**Rubin (2013) [22]**	**Lofgren (2014) [23]**	**Yakob (2014) [24]**	**Lanzas (2014) [25]**	**Codella (2015) [26]**
**5-OUTCOME OF MODEL**										
**21**	Have clear descriptions of the outcomes been provided?	L-M	M-H	M-H	M-H	M-H	M-H	M-H	M-H	M
**22**	Have the baseline values (before any interventions) of outcomes been clearly reported?	N/A	H	H	M	H	H	M	H	M
**23**	For the principal findings of the study, are the outcomes reported numerically or are they presented in such a way that allows data extraction?	N/A	H	H	L	M-H	H	L	H	M-H
**24**	Have the units of measurements used for the outcomes been clearly defined?	N/A	M-H	H	M-H	H	H	M-H	H	L
**25**	Has a graphical representation of the transmission dynamics over time been reported (e.g., incidence and/or prevalence of infection/disease)?	N/A	H	L	H	L	H	L	L	L
**6-UNCERTAINTY ANALYSES AND SENSITIVITY ANALYSES**										
**26**	Have stochastic (first-order) uncertainties been assessed/reported?	N/A	H	L	L	H	H	L	H	H
**27**	Have parametric uncertainties been assessed/reported?	N/A	N/A	M-H	L	M	L	L	M	M
**28**	Have structural uncertainties been assessed/reported?	N/A	L	L	L	L	L	L	L	L
**29**	Have calibration uncertainties been assessed/reported?	N/A	H	N/A	N/A	N/A	N/A	N/A	N/A	H
**30**	Have sensitivity analyses been assessed/reported?	N/A	M	H	H	H	L	M	H	H
**7-VALIDATION AND THE QUALITY OF DOCUMENTATION**										
**31**	Has the study reported a model validation (face; internal; cross; external; predictive-validity)?	N/A	L	L	L	H	L	L	L	H
**32**	Have limitations and strengths of the model been acknowledged?	M	M-H	H	H	H	H	H	L-M	H
**33**	Is complementary information provided (appendix, supplementary file...) or is there mention of reference of technical documentations available on request?	N/A	L	H	H	H	H	L	M-H	L
**34**	Are there graphical descriptions of some model concepts?	M-H	M-H	M	M-H	H	M-H	M-H	L-M	H
**35**	Does the study contains no errors, inconsistencies or missing information?	N/A	M-H	M	L-M	M-H	M	M	M-H	L-M

Table S2.2: Rating scale for the quality of reporting

NOT APPLICABLE	LOW	LOW-MEDIUM	MEDIUM	MEDIUM-HIGH	HIGH
N/A	L	L-M	M	M-H	H

References

[1] US Environmental Protection Agency, Science Policy Council. White Paper on the Nature and Scope of Issues on Adoption of Model Use Acceptability Guidance; 1999 [cited 2016 Mar 18]. Available from: http://www.epa.gov/sites/production/files/2015-02/documents/whitepaper_1999.pdf. pages (p.↑1), (p.↑3)

[2] Committee on Models in the Regulatory Decision Process, Board on Environmental Studies and Toxicology, Division on Earth and Life Studies, National Research Council. Models in Environmental Regulatory Decision Making. National Academies Press; 2007 [cited 2016 Mar 15]. Availaible from: http://www2.lbl.gov/today/2007/Jun/22-Fri/Models-report.pdf doi: 10.17226/11972. pages (p.↑1), (p.↑2), (p.↑3)

[3] Weinstein MC, O'Brien B, Hornberger J, Jackson J, Johannesson M, McCabe C, et al. Principles of Good Practice for Decision Analytic Modeling in Health-Care Evaluation: Report of the ISPOR Task Force on Good Research Practices--Modeling Studies. Value Health. 2003;6(1):9–17. doi: 10.1046/j.1524-4733.2003.00234.x PMID: 12535234. pages (p.↑1), (p.↑2), (p.↑3)

[4] Kopec J, Fines P, Manuel D, Buckeridge D, Flanagan W, Oderkirk J, et al. Validation of population-based disease simulation models: a review of concepts and methods. BMC Public Health. 2010;10(1):710. doi: 10.1186/1471-2458-10-710 PMID: 21087466 PubMed Central PMCID: PMC3001435. pages (p.↑1), (p.↑2), (p.↑3)

[5] Briggs AH, Weinstein MC, Fenwick EAL, Karnon J, Sculpher MJ, Paltiel AD. Model Parameter Estimation and Uncertainty: A Report of the ISPOR-SMDM Modeling Good Research Practices Task Force-6. Value Health. 2012;15(6):835–842. doi: 10.1016/j.jval.2012.04.014 PMID: 22999133. pages (p.↑1), (p.↑2), (p.↑3)

[6] Eddy DM, Hollingworth W, Caro JJ, Tsevat J, McDonald KM, Wong JB. Model Transparency and Validation: A Report of the ISPOR-SMDM Modeling Good Research Practices Task Force-7. Med Decis Making. 2012;32(5):733–743. doi: 10.1177/0272989x12454579 PMID: 22999134. pages (p.↑1), (p.↑3)

[7] Roberts M, Russell LB, Paltiel AD, Chambers M, McEwan P, Krahn M. Conceptualizing a Model: A Report of the ISPOR-SMDM Modeling Good Research Practices Task Force-2. Value Health. 2012;15(6):804–811. doi: 10.1016/j.jval.2012.06.016 PMID: 22999129 PubMed Central PMCID: PMC4207095. pages (p.↑1), (p.↑2), (p.↑3)

[8] Peñaloza Ramos MC, Barton P, Jowett S, Sutton AJ. A Systematic Review of Research Guidelines in Decision-Analytic Modeling. Value Health. 2015;18(4):512–529. doi: 10.1016/j.jval.2014.12.014 PMID: 26091606. pages (p.↑1), (p.↑2), (p.↑3)

[9] Pitman R, Fisman D, Zaric GS, Postma M, Kretzschmar M, Edmunds J, et al. Dynamic Transmission Modeling: A Report of the ISPOR-SMDM Modeling Good Research Practices Task Force-5. Value Health. 2012;15(6):828–834. doi: 10.1016/j.jval.2012.06.011 PMID: 22999132. pages (p.↑1), (p.↑2), (p.↑3)

[10] Weinstein MC, Toy EL, Sandberg EA, Neumann PJ, Evans JS, Kuntz KM, et al. Modeling for Health Care and Other Policy Decisions: Uses, Roles, and Validity. Value Health. 2001;4(5):348–361. doi: 10.1046/j.1524-4733.2001.45061.x PMID: 11705125. pages (p.↑1), (p.↑3)

[11] Philips Z, Ginnelly L, Sculpher M, Claxton K, Golder S. Review of guidelines for good practice in decision-analytic modelling in health technology assessment. Health Technol Assess. 2004;8(36):172. doi: 10.3310/hta8360 PMID: 15361314. pages (p.↑1), (p.↑2), (p.↑3)

[12] Philips Z, Bojke L, Sculpher M, Claxton K, Golder S. Good Practice Guidelines for Decision-Analytic Modelling in Health Technology Assessment. Pharmacoeconomics. 2006;24(4):355–371. doi: 10.2165/00019053-200624040-00006 PMID: 16605282. pages (p.↑1), (p.↑2), (p.↑3)

[13] Kuntz K, Sainfort F, Butler M, Taylor B, Kulasingam S, Gregory S, et al. Decision and Simulation Modeling in Systematic Reviews. Agency for Healthcare Research and Quality (US); 2013 [cite 2016 Mar 15]. PMID: 23534078. Available from: http://www.ncbi.nlm.nih.gov/books/NBK127482/. pages (p.↑1), (p.↑2), (p.↑3)

[14] Husereau D, Drummond M, Petrou S, Carswell C, Moher D, Greenberg D, et al. Consolidated Health Economic Evaluation Reporting Standards (CHEERS)-Explanation and Elaboration: A Report of the ISPOR Health Economic Evaluation Publication Guidelines Good Reporting Practices Task Force. Value Health. 2013;16(2):231–250. doi: 10.1016/j.jval.2013.02.002 PMID: 23538175. pages (p.↑1), (p.↑2), (p.↑3)

[15] Jaime Caro J, Eddy DM, Kan H, Kaltz C, Patel B, Eldessouki R, et al. Questionnaire to Assess Relevance and Credibility of Modeling Studies for Informing Health Care Decision Making: An ISPOR-AMCP-NPC Good Practice Task Force Report. Value Health. 2014;17(2):174–182. doi: 10.1016/j.jval.2014.01.003 PMID: 24636375. pages (p.↑1), (p.↑2), (p.↑3)

[16] The US Environmental Protection Agency, Council of Regulatory Environmental Modeling, Office of the Science Advisor. Guidance on the Development, Evaluation, and Application of Environmental Models; 2009 [cited 2015 Mar 15]. Available from: http://nepis.epa.gov/Exe/ZyPDF.cgi?Dockey=P1003E4R.PDF. pages (p.↑2), (p.↑3)

[17] Thacker BH, Doebling SW, Hemez FM, Anderson MC, Pepin JE, Rodriguez EA. Concepts of Model Verification and Validation. Los Alamos National Laboratory; 2004 [cited 2016 Mar 17]. Available from: http://www.ltas-vis.ulg.ac.be/cmsms/uploads/File/LosAlamos_VerificationValidation.pdf. pages (p.↑3)

[18] Starr JM, Campbell A. Mathematical modeling of *Clostridium difficile* infection. Clinical Microbiol and Infect. 2001;7(8):432–437. doi: 10.1046/j.1198-743x.2001.00291.x PMID: 11591207. pages (p.↑3), (p.↑4), (p.↑5)

[19] Starr JM, Campbell A, Renshaw E, Poxton IR, Gibson GJ. Spatio-temporal stochastic modelling of *Clostridium difficile*. J Hosp Infect. 2009;71(1):49–56. doi: 10.1016/j.jhin.2008.09.013 PMID: 19013677. pages (p.↑3), (p.↑4), (p.↑5)

[20] Lanzas C, Dubberke ER, Lu Z, Reske KA, Gröhn YT. Epidemiological Model for *Clostridium difficile* Transmission in Healthcare Settings. Infect Control Hosp Epidemiol. 2011;32(6):553–561. doi: 10.1086/660013 PMID: 21558767 PubMed Central PMCID: PMC3645005. pages (p.↑3), (p.↑4), (p.↑5)

[21] Yakob L, Riley T, Paterson D, Clements A. *Clostridium difficile* exposure as an insidious source of infection in healthcare settings: an epidemiological model. BMC Infect Dis. 2013;13(1):376. doi: 10.1186/1471-2334-13-376 PMID: 23947736 PubMed Central PMCID: PMC3751620. pages (p.↑3), (p.↑4), (p.↑5)

[22] Rubin MA, Jones M, Leecaster M, Khader K, Ray W, Huttner A, et al. A Simulation-Based Assessment of Strategies to Control *Clostridium Difficile* Transmission and Infection. PLoS One;8(11):e80671. doi: 10.1371/journal.pone.0080671 PMID: 24278304 PubMed Central PMCID: PMC3836736. pages (p.↑3), (p.↑4), (p.↑5)

[23] Lofgren ET, Moehring RW, Anderson DJ, Weber DJ, Fefferman NH. A Mathematical Model to Evaluate the Routine Use of Fecal Microbiota Transplantation to Prevent Incident and Recurrent *Clostridium difficile* Infection. Infect Control Hosp Epidemiol. 2014;35(1):18–27. doi: 10.1086/674394 PMID: 24334794 PubMed Central PMCID: PMC3977703. pages (p.↑3), (p.↑4), (p.↑5)

[24] Yakob L, Riley TV, Paterson DL, Marquess J, Clements ACA. Assessing control bundles for *Clostridium difficile*: a review and mathematical model. Emerg Microbes Infect. 2014;3:e43. doi: 10.1038/emi.2014.43 PMID: 26038744 PubMed Central PMCID: PMC4078791. pages (p.↑3), (p.↑4), (p.↑5)

[25] Lanzas C, Dubberke ER. Effectiveness of Screening Hospital Admissions to Detect Asymptomatic Carriers of *Clostridium difficile*: A Modeling Evaluation. Infect Control Hosp Epidemiol. 2014;35(08):1043–1050. doi: 10.1086/677162 PMID: 25026622. pages (p.↑3), (p.↑4), (p.↑5)

[26] Codella J, Safdar N, Heffernan R, Alagoz O. An Agent-based Simulation Model for *Clostridium difficile* Infection Control. Med Decis Making. 2015;35(2):211–29. doi: 10.1177/0272989X14545788 PMID: 25112595 PubMed Central PMCID: PMC4311776. pages (p.↑3), (p.↑4), (p.↑5)
