# Supplementary material for: Mathematical Modeling of the Transmission Dynamics of Clostridium difficile Infection and Colonization in Healthcare Settings: A Systematic Review
Source: PLoS One. 2016 Sep 30;11(9):e0163880. doi: 10.1371/journal.pone.0163880 (PMC5045168; doi:10.1371/journal.pone.0163880)
Supplement: S3 Appendix — (PDF) [file pone.0163880.s004.pdf]

S3 Appendix. Analysis of the natural history representations and transmission dynamics

Natural history refers to the progression of the disease across health states in an individual over time. It can be represented schematically by a set of mutually-exclusive health states (compartments) linked by arrows indicating the possible transitions (progression/regression) between the health states. In a mathematical model, these transitions are parameterized and dynamics is modeled either by deterministic or stochastic techniques. In a model framework, the natural history representation refers to the conceptual sets of health states and health state transitions that are used mathematically to represent infectious disease dynamics in a population. The common compartmental models of infectious diseases are generally based upon the frameworks developed by Ross [1], Kermack and McKendrick [2] from which we find the basic SIR model (Susceptible-Infectious-Recovered). Some more complex models [3, 4] can involve other health states and their structure is usually based upon specific assumptions.

Model template for the analysis of the natural history representations

To compare all natural histories reported in the mathematical models reviewed for the transmission dynamics of *C. difficile* in healthcare settings, we have analyzed, within each study, the natural history representation and the transmission dynamics using a compartmental epidemic model template. Our template divides the natural history into six possible health states (S-E-A-I-R-D: Susceptible-Exposed-Asymptomatic-Infectious-Resistant-Deceased) as represented in **Fig S3.1**. Thus, our model template consists of an extension of the basic SIR model for which, three additional compartments are included (E-A-D). This analysis is intended to synthesize the natural history representations reported in the reviewed models.

Specifically, this analysis aims to:

1) clearly identify every health state and every transition defined in the natural history representations;
2) stratify every health state, according to the modeled population characteristics;
3) isolate and identify the transmission pathways included in the models;
4) identify patient flow included in the models.

Health states identified in models are first assigned to a possible health state (S-E-A-I-R-D) defined by our template. Then, we analyze the following components, if included: 1) transitions between the health states; 2) stratification of the health states; 3) transmission pathways; and 4) patient flow (admissions and discharges from the healthcare setting). Transmission can be done indirectly from the environment or from the healthcare workers (HCWs) and visitors. When they are modeled, the HCWs or the visitors are assigned to one of two states: contaminated or non-contaminated. It is assumed that HCWs and visitors cannot be infected (the ingestion of spores or the colonization are not possible), but they participate indirectly in the transmission by increasing patient exposure to the pathogen. This kind of interaction between the HCWs/visitors and the natural history representation is referred as a ”vector-host” component of transmission (Ross-MacDonald [1, 5]).

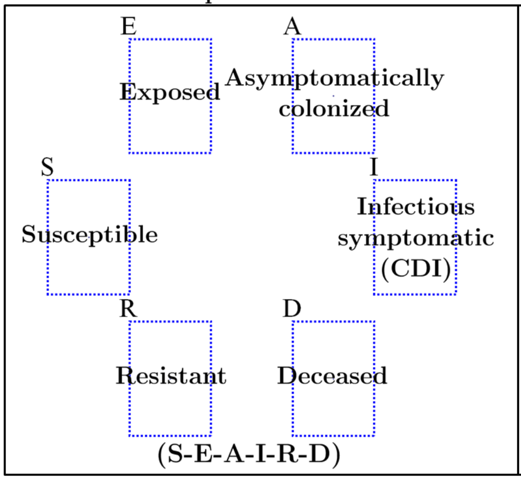

Definitions of the template health states

- **S (Susceptible) :** refers to the individuals that have no resistance/immunity to the infection. They can become infected (i.e., they can ingest the spores of *C. difficile* or be colonized by the pathogen) from contacts with infectious individuals (asymptomatic and symptomatic).
- **E (Exposed) :** refers to the individuals infected after their ingestion of *C. difficile* spores. The individuals are not infectious yet. This health state represents a latent period where the spores germinate to form vegetative cells.
- **A (Asymptomatically colonized) :** refers to the bacterial colonization process of the intestinal tracts of individuals. The individuals are infectious, but do not present any symptom.
- **I (Infectious symptomatic (CDI)) :** refers to the symptomatic manifestations of the disease (usually diarrhea) in individuals.
- **R (Resistant) :** refers to the individuals having a natural resistance or an acquired immunity to the infection.
- **D (Deceased) :** refers to the individuals who died from *C. difficile* infection.

Fig S3.1: The epidemic model template of health states (S-E-A-I-R-D) used to analyze the natural history representations and the transmission dynamics of the models. We included a brief description of each health state in the context of *C. difficile* infection. All the health states reported in the models can be associated with one of these definitions. Indeed, we assumed that each of the health states (S-E-A-I-R-D) defined can be further stratified to allow the inclusion of the population heterogeneities. Also, in our definitions, we used the general term "infection" to refer to the acquisition and to the colonization of *C. difficile* in an individual, and "*Clostridium difficile* infection (CDI)" to refer to the symptomatic stage of the disease.

In our analysis framework we use two diagrams: one to describe the natural history representation (left diagram), and another to present the transmission/exposure pathways alongside with the flow of individuals arriving or departing in each health state (right diagram). We present in **Fig S3.2** an example of this kind of analysis applied to the basic SIR model with demography (births and deaths).

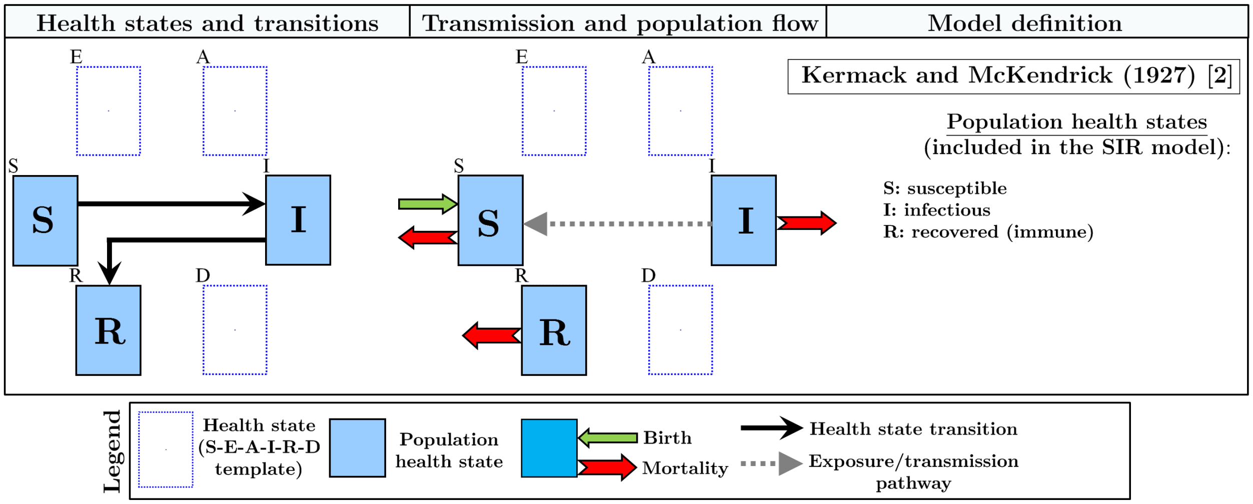

Fig S3.2: An example of our epidemic model template applied to the basic SIR model with demography.

The two diagrams contain the following information:

Left diagram: health states and health state transitions. The diagram presents a conceptual picture of the natural history of the disease, according to our epidemic model template (**Fig S3.1**). Each of the six health states (S-E-A-I-R-D) of the model template (the dashed blue rectangles) can be filled by one or many health states (the blue filled boxes) that are defined in a model examined. The black arrows represent transitions (state change) between the health states and are parameterized accordingly. For aggregated population models, the population characteristics included in the natural history representation, can be expressed by additional stratifications within each health state of the template (independent compartments). These additional stratifications are specific to each study and depend on the model assumptions. For individual-based models, these stratifications do not necessarily need to be included in the natural history representation, as the model can track individuals.

Right diagram: transmission pathways and patient flow. The diagram presents the links (gray dashed arrows) that conceptually represent the transmission (direct or indirect pathways) of the pathogen to (or from) a human/non-human reservoir of infection. These links can also represent a contamination process. In every case, one transmission path needs to be linked to the susceptible individuals in order for infection events to occur. Furthermore, we have represented the flow of individuals in each health state (the system is usually open) using green arrows (admissions/discharges in the context of hospital-associated *C. difficile* infections). The mortality, when it's not included as a separated health state, is presented by a red arrow exiting the health states.

Results and data synthesis

Seven studies [6, 7, 8, 9, 10, 11, 12] provided specific information on their natural history representation. The agent-based model presented in Lanzas *et al.* (2014) [13] was based on the natural history representation developed previously by the same authors (Lanzas *et al.* (2011) [7]). Also, we identified that the conceptual model developed in Starr *et al.* (1997) [14] for nosocomial infections of *C. difficile* was the basis for the models developed in the following studies in Starr *et al.* (2001-2009) [15, 6]. Thus, six independent natural history representations have been retrieved.

The data regarding the natural history representation and the transmission pathways were extracted from the studies by using the information provided directly in the articles (text, tables, conceptual diagrams or logic diagrams (agent-based models)) or in the supplementary files or by contacting the authors of the studies. When possible, we have also reported the notation used in each study to describe the health states. Otherwise, we have defined the notation. All the data retrieved were summarized and are presented in **Fig S3.3** and **Fig S3.4**. Then, the graphical analysis of these representations has allowed us to synthesize the natural history representations of the models examined into four tables. These tables included the following information:

1. summary of health state transitions (**Table S3.1**);
2. health state stratifications according to patient characteristics (**Table S3.2**);
3. contamination/transmission pathways (**Table S3.3**);
4. patient flow (admissions and discharges of patients from the healthcare setting which are among the health states of the model template S-E-A-I-R-D) (**Table S3.4**).

By this kind of analysis and by the use of these two diagrams presented in **Fig S3.3** and **Fig S3.4**, it is possible to compare the general structures of the natural history representations across the studies.

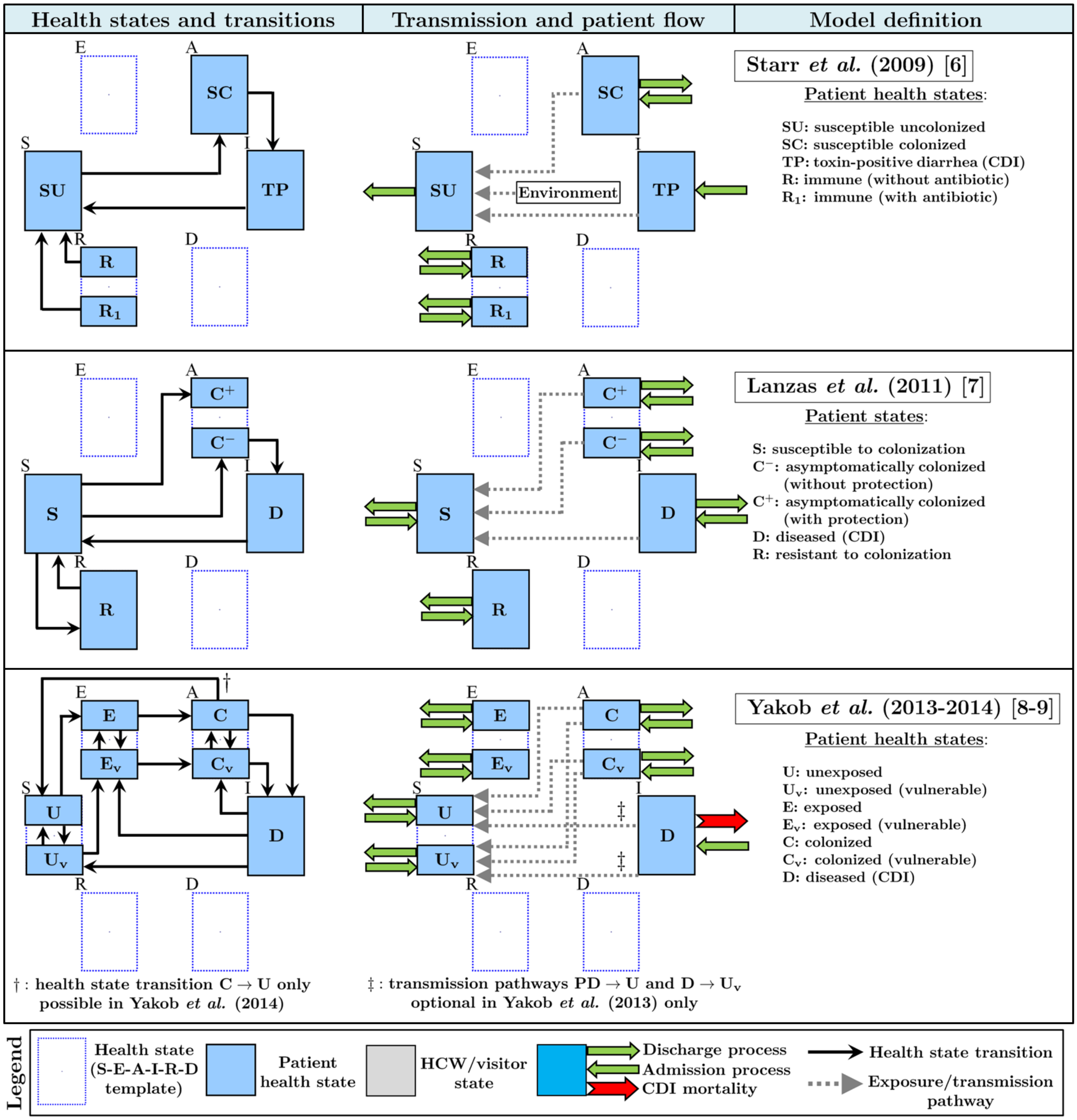

Fig S3.3: Natural history representations and transmission dynamics of mathematical models for *C. difficile* infections in healthcare settings. The models represented on this page (Starr *et al.* (2009) [6], Lanzas *et al.* (2011) [7], and Yakob *et al.* (2013-2014) [8, 9]) are those that didn't include the healthcare workers and visitors as vector-host components of indirect transmission.

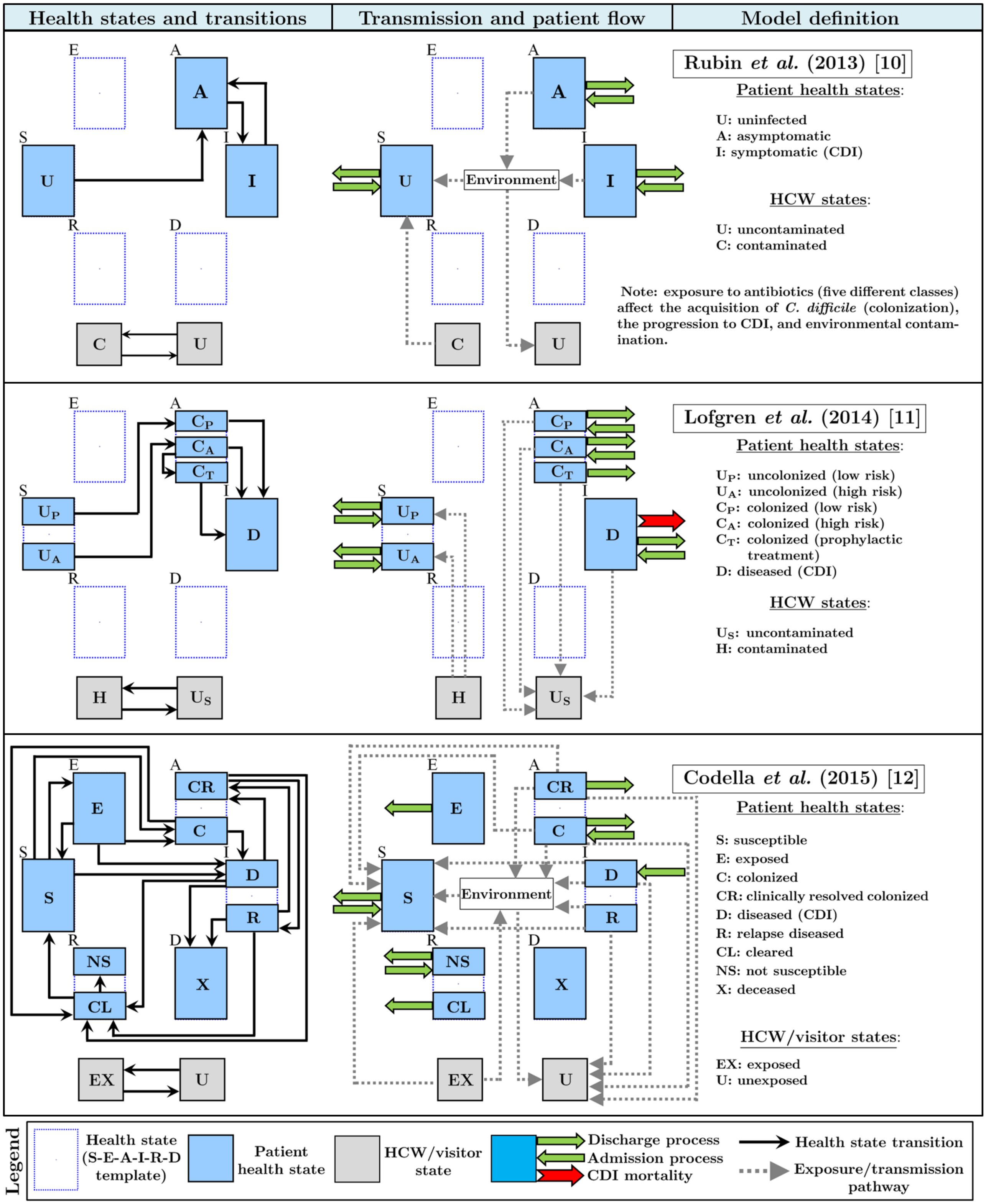

Fig S3.4: Natural history representations and transmission dynamics of mathematical models for *C. difficile* infections in healthcare settings. The models represented on this page (Rubin *et al.* (2013) [10], Lofgren *et al.* (2014) [11], and Codella *et al.* (2015) [12]) are those that included the healthcare workers and visitors as vector-host components of indirect transmission.

Tables S3.1: Possible health state transitions for each model. The existence of a health state transition is represented by blue cells with a check mark ($\checkmark$). The transitions are designated from a row **(current health state)** to a column **(next health state)**. The self-transitions to the same current health state (e.g., $S \to S$) are not represented, but are also possible.

a) Starr *et al.* (2009) [6]

Current health state ↱ Next health state	SU	SC	TP	R	R_1
SU	–	$\checkmark$	–	–	–
SC	–	–	$\checkmark$	–	–
TP	$\checkmark$	–	–	–	–
R	$\checkmark$	–	–	–	–
R_1	$\checkmark$	–	–	–	–

b) Lanzas *et al.* (2011) [7]

Current health state ↱ Next health state	S	C^-	C^+	D	R
S	–	$\checkmark$	$\checkmark$	–	$\checkmark$
C^-	–	–	–	$\checkmark$	–
C^+	–	–	–	–	–
D	$\checkmark$	–	–	–	–
R	$\checkmark$	–	–	–	–

c) Yakob *et al.* (2013-2014) [8, 9]

Current health state ↱ Next health state	U	U_v	E	E_v	C	C_v	D
U	–	$\checkmark$	$\checkmark$	–	–	–	–
U_v	$\checkmark$	–	–	$\checkmark$	–	–	–
E	–	–	–	$\checkmark$	$\checkmark$	–	–
E_v	–	–	$\checkmark$	–	–	$\checkmark$	–
C	($\checkmark$)	–	–	–	–	$\checkmark$	$\checkmark$
C_v	–	–	–	–	$\checkmark$	–	$\checkmark$
D	–	$\checkmark$	–	–	–	$\checkmark$	–

($\checkmark$): health state transition $C \to U$ only possible in Yakob *et al.* (2014)

d) Rubin *et al.* (2013) [10]

Current health state ↱ Next health state	U	A	I
U	–	$\checkmark$	–
A	–	–	$\checkmark$
I	–	$\checkmark$	–

e) Lofgren *et al.* (2014) [11]

Current health state ↱ Next health state	U_P	U_A	C_P	C_A	C_T	D
U_P	–	–	$\checkmark$	–	–	–
U_A	–	–	–	$\checkmark$	–	–
C_P	–	–	–	–	–	$\checkmark$
C_A	–	–	–	–	$\checkmark$	$\checkmark$
C_T	–	–	–	–	–	–
D	–	–	–	–	–	–

f) Codella *et al.* (2015) [12]

Current health state ↱ Next health state	S	E	C	CR	D	R	CL	NS	X
S	–	$\checkmark$	$\checkmark$	–	$\checkmark$	–	–	–	–
E	$\checkmark$	–	$\checkmark$	–	$\checkmark$	–	–	–	–
C	–	–	–	–	$\checkmark$	–	$\checkmark$	–	–
CR	–	–	–	–	–	$\checkmark$	$\checkmark$	–	–
D	–	–	–	$\checkmark$	–	–	$\checkmark$	–	$\checkmark$
R	–	–	–	–	–	–	$\checkmark$	–	$\checkmark$
CL	$\checkmark$	–	–	–	–	–	–	$\checkmark$	–
NS	–	–	–	–	–	–	–	–	–
X	–	–	–	–	–	–	–	–	–

$\checkmark$: health state transition is allowed (Current health state) $\to$ (Next health state)
–: health state transition is not allowed (Current health state) $\nrightarrow$ (Next health state)

Table S3.2: Stratifications of each health state of the template (S-E-A-I-R-D) according to each model.

Model:	S	E	A	I	R	D	Model type
**Starr *et al.* [6]**	SU	–	SC	TP	R, R_1	–	**S-A-I-R**
**Lanzas *et al.* [7]**	S	–	C^-, C^+	D	R	–	**S-A-I-R**
**Yakob *et al.* [8, 9]**	U, U_v	E, E_v	C, C_v	D	–	–	**S-E-A-I**
**Rubin *et al.* [10]**	U	–	A	I	–	–	**S-A-I**
**Lofgren *et al.* [11]**	U_P, U_A	–	C_P, C_A, C_T	D	–	–	**S-A-I**
**Codella *et al.* [12]**	S	E	C, CR	D, R	CL, NS	X	**S-E-A-I-R-D**

Starr *et al.*: **SU**: susceptible uncolonized; **SC**: susceptible colonized; **TP**: toxin-positive diarrhea; **R**, $\mathbf{R_1}$:immune (without/with exposure to antibiotics). **Lanzas *et al.***: **S**: susceptible (exposure to antibiotics); $\mathbf{C^-}$: asymptomatically colonized (without immune protection); $\mathbf{C^+}$: asymptomatically colonized (with immune protection); **D**: diseased (CDI); **R**: resistant. **Yakob *et al.***: **U**: unexposed; **E**: exposed; **C**: colonized; **D**: diseased (CDI); (**subscript** $_v$: vulnerable (exposure to antibiotics)). **Rubin *et al.***: **U**: uninfected; **A**: asymptomatic; **I**: symptomatic (CDI). **Lofgren *et al.***: **U**: uncolonized; **C**: colonized; **D**; diseased (CDI); (**subscripts** $_P$/$_A$: exposure to low/high risk antibiotics respectively); (**subscript** $_T$: prophylactic treatment). **Codella *et al.***: **S**: susceptible; **E**: exposed; **C**: colonized; **CR**: clinically resolved colonized; **D**: diseased CDI; **R**: relapse diseased; **CL**: cleared; **NS**: not susceptible; **X**: deceased.

Table S3.3: Transmission/contamination pathways for the models included.

**Transmission pathways:**	**Model:**	**Starr *et al.* [6]**	**Lanzas *et al.* [7]**	**Yakob *et al.* [8, 9]**	**Rubin *et al.* [10]**	**Lofgren *et al.* [11]**	**Codella *et al.* [12]**
Symptomatic to	susceptible (D)	✓	✓	(✓)[a]	–	–	✓
	environment (I)	–	–	–	✓	**(partial)**[b]	✓
	HCW/visitor (I)	–	–	–	–	✓	✓
Asymptomatic to	susceptible (D)	✓	✓	✓	–	–	✓
	environment (I)	–	–	–	✓	**(partial)**[b]	✓
	HCW/visitor (I)	–	–	–	–	✓	✓
Environment to	susceptible (I)	✓	–	–	✓	**(partial)**[b]	✓
	HCW/visitor (I)	–	–	–	✓	**(partial)**[b]	✓
HCW/visitor to	susceptible (I)	–	–	–	✓	✓	✓
	environment (I)	–	–	–	–	–	✓
**Direct transmission pathways**		✓	✓	✓	–	–	✓
**Indirect transmission pathways**		✓	–	–	✓	✓	✓

✓/–: pathways allowed/not allowed; **(I):** indirect transmission pathway; **(D):** direct transmission pathway; **HCW:** healthcare worker.
[a] Transmission pathways denoted by (✓) are optional in the first version of the model only (Yakob *et al.* (2013) [8]).
[b] Transmission pathways denoted by (partial) are not explicitly modeled (included in the contact rate parameters between HCWs and patients). These pathways refer to environmental contamination and to transmission from the environment.

Table S3.4: Patient flow (admissions and discharges) for the models included.

a) Patient admission

Model:	S	E	A	I	R	D
**Starr *et al.* [6]**	–	–	✓	✓	✓	–
**Lanzas *et al.* [7]**	✓	–	✓	✓	✓	–
**Yakob *et al.* [8, 9]**	✓	✓	✓	✓	–	–
**Rubin *et al.* [10]**	✓	–	✓	✓	–	–
**Lofgren *et al.* [11]**	✓	–	✓	✓	–	–
**Codella *et al.* [12]**	✓	–	✓	✓	✓	–

✓/–: the process is possible/not possible

b) Patient discharge

Model:	S	E	A	I	R	D
**Starr *et al.* [6]**	✓	–	✓	–	✓	–
**Lanzas *et al.* [7]**	✓	–	✓	✓	✓	–
**Yakob *et al.* [8, 9]**	✓	✓	✓	✓	–	–
**Rubin *et al.* [10]**	✓	–	✓	✓	–	–
**Lofgren *et al.* [11]**	✓	–	✓	✓	–	–
**Codella *et al.* [12]**	✓	✓	✓	–	✓	–

✓/–: the process is possible/not possible

Natural history representations of *C. difficile* infection related to reviewed models

We have identified two conceptual natural history representations used by the models by reviewing references of selected studies. These representations were purely conceptual, that is to say that they included no parameter, but only definitions of health states, health state transitions and transmission pathways. We report in **Fig S3.5** (upper figure) the conceptual model developed by Starr *et al.* (1997) [14] used to represent the epidemiology of nosocomial *C. difficile* infections. This conceptual model has laid the basis for the following studies in Starr *et al.* (2001-2009) [15, 6]. Also, in **Fig S3.5** (lower figure), we report the natural history representation of community-associated *C. difficile* infection (CA-CDI) developed by Otten *et al.* (2010) [16]. A modified version of this framework was used in the study of Codella *et al.* (2015) [12].

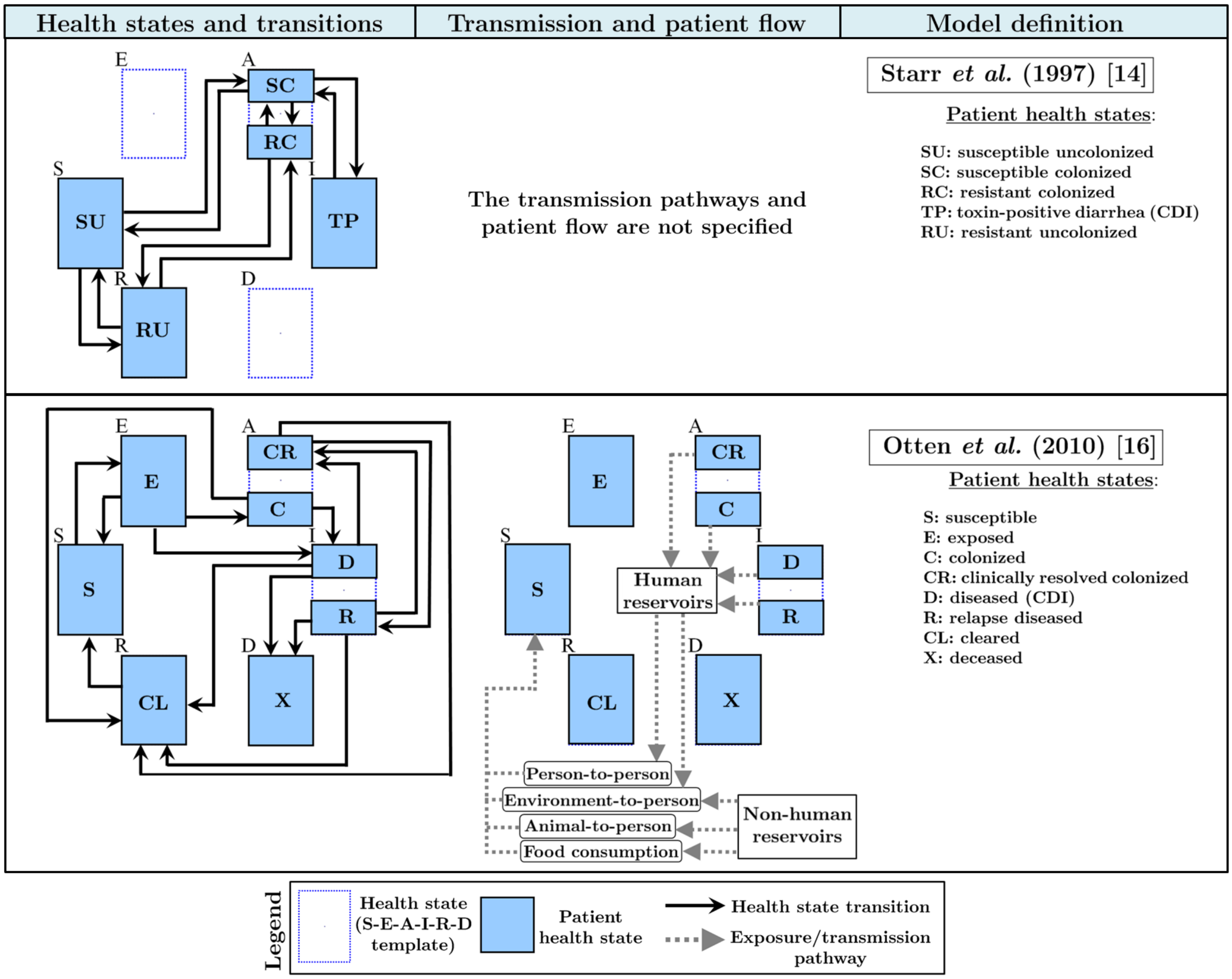

Fig S3.5: Natural history representations of *C. difficile* infection related to reviewed models. The upper figure shows the conceptual model for the natural history of nosocomial *C. difficile* infections in Starr *et al.* (1997) [14]. The lower figure shows the conceptual model for the natural history of community-associated *C. difficile* infections in Otten *et al.* (2010) [16].

References

[1] Ross R. The prevention of malaria. Dutton; 1910. pages (p.↑1)

[2] Kermack WO, McKendrick AG. A Contribution to the Mathematical Theory of Epidemics. Proc R Soc A. 1927 [cited 2016 Mar 21];115(772):700–721. doi: 10.1098/rspa.1927.0118. Available from: http://rspa.royalsocietypublishing.org/content/115/772/700. pages (p.↑1)

[3] Brauer F, van den Driessche P, Wu J. Mathematical Epidemiology. Lecture Notes in Mathematics. Springer Berlin Heidelberg; 2008 [cited 2016 Mar 23]. doi: 10.1007/978-3-540-78911-6. Available from: http://link.springer.com/book/10.1007%2F978-3-540-78911-6. pages (p.↑1)

[4] Brauer F, Castillo-Chavez C. Mathematical Models in Population Biology and Epidemiology. Texts in Applied Mathematics. Springer New York; 2012 [cited 2016 Mar 23]. doi: 10.1007/978-1-4614-1686-9. Available from: http://link.springer.com/book/10.1007/978-1-4614-1686-9. pages (p.↑1)

[5] McDonald G. The epidemiology and control of malaria. Oxford, UK: Oxford University Press; 1957. pages (p.↑1)

[6] Starr JM, Campbell A, Renshaw E, Poxton IR, Gibson GJ. Spatio-temporal stochastic modelling of *Clostridium difficile*. J Hosp Infect. 2009;71(1):49–56. doi: 10.1016/j.jhin.2008.09.013 PMID: 19013677. pages (p.↑3), (p.↑4), (p.↑6), (p.↑7), (p.↑8)

[7] Lanzas C, Dubberke ER, Lu Z, Reske KA, Gröhn YT. Epidemiological Model for *Clostridium difficile* Transmission in Healthcare Settings. Infect Control Hosp Epidemiol. 2011;32(6):553--561. doi: 10.1086/660013 PMID: 21558767 PubMed Central PMCID: PMC3645005. pages (p.↑3), (p.↑4), (p.↑6), (p.↑7)

[8] Yakob L, Riley T, Paterson D, Clements A. *Clostridium difficile* exposure as an insidious source of infection in healthcare settings: an epidemiological model. BMC Infect Dis. 2013;13(1):376. doi: 10.1186/1471-2334-13-376 PMID: 23947736 PubMed Central PMCID: PMC3751620. pages (p.↑3), (p.↑4), (p.↑6), (p.↑7)

[9] Yakob L, Riley TV, Paterson DL, Marquess J, Clements ACA. Assessing control bundles for *Clostridium difficile*: a review and mathematical model. Emerg Microbes Infect. 2014;3:e43. doi: 10.1038/emi.2014.43 PMID: 26038744 PubMed Central PMCID: PMC4078791. pages (p.↑3), (p.↑4), (p.↑6), (p.↑7)

[10] Rubin MA, Jones M, Leecaster M, Khader K, Ray W, Huttner A, et al. A Simulation-Based Assessment of Strategies to Control *Clostridium Difficile* Transmission and Infection. PLoS One;8(11):e80671. doi: 10.1371/journal.pone.0080671 PMID: 24278304 PubMed Central PMCID: PMC3836736. pages (p.↑3), (p.↑5), (p.↑6), (p.↑7)

[11] Lofgren ET, Moehring RW, Anderson DJ, Weber DJ, Fefferman NH. A Mathematical Model to Evaluate the Routine Use of Fecal Microbiota Transplantation to Prevent Incident and Recurrent *Clostridium difficile* Infection. Infect Control Hosp Epidemiol. 2014;35(1):18–27. doi: 10.1086/674394 PMID: 24334794 PubMed Central PMCID: PMC3977703. pages (p.↑3), (p.↑5), (p.↑6), (p.↑7)

[12] Codella J, Safdar N, Heffernan R, Alagoz O. An Agent-based Simulation Model for *Clostridium difficile* Infection Control. Med Decis Making. 2015;35(2):211–29. doi: 10.1177/0272989X14545788 PMID: 25112595 PubMed Central PMCID: PMC4311776. pages (p.↑3), (p.↑5), (p.↑6), (p.↑7), (p.↑8)

[13] Lanzas C, Dubberke ER. Effectiveness of Screening Hospital Admissions to Detect Asymptomatic Carriers of *Clostridium difficile*: A Modeling Evaluation. Infect Control Hosp Epidemiol. 2014;35(08):1043–1050. doi: 10.1086/677162 PMID: 25026622. pages (p.↑3)

[14] Starr JM, Rogers TR, Impallomeni M. Hospital-acquired *Clostridium difficile* diarrhoea and herd immunity. Lancet. 1997;349(9049):426–8. doi: 10.1016/S0140-6736(97)80053-0 PMID: 9033485. pages (p.↑3), (p.↑8)

[15] Starr JM, Campbell A. Mathematical modeling of *Clostridium difficile* infection. Clinical Microbiol and Infect. 2001;7(8):432–437. doi: 10.1046/j.1198-743x.2001.00291.x PMID: 11591207. pages (p.↑3), (p.↑8)

[16] Otten AM, Reid-Smith RJ, Fazil A, Weese JS. Disease transmission model for community-associated *Clostridium difficile* infection. Epidemiol Infect. 2010;138(06):907–914. doi: 10.1017/S0950268809991646 PubMed PMID: 20092667. pages (p.↑8)
